# Action of the Metalloproteinases in Gonadal Remodeling during Sex Reversal in the Sequential Hermaphroditism of the Teleostei Fish *Synbranchus marmoratus* (Synbranchiformes: Synbranchidae)

**DOI:** 10.3390/cells7050034

**Published:** 2018-04-24

**Authors:** Talita Sarah Mazzoni, Fabiana Laura Lo Nostro, Fernanda Natália Antoneli, Irani Quagio-Grassiotto

**Affiliations:** 1 Department of Cell and Development Biology, Institute of Biomedical Sciences, Federal University of Alfenas (UNIFAL), Gabriel Monteiro da Silva 700, 37130-001 Alfenas-MG, Brazil; talitasarah@yahoo.com.br; 2 Department of Morphology, Botucatu Biosciences Institute, State University of São Paulo (UNESP), Prof. Dr. Antonio Celso Wagner Zanin 250, 18618-689 Botucatu-SP, Brazil; 3 Department of Biodiversity and Experimental Biology, Faculty of Exact and Natural Sciences, Ciudad Universitaria, Lab. 4 y 78; Piso 4to., Pabellón 2, Int. Güiraldes 2160, C1428EGA Buenos Aires, Argentina; fabi@bg.fcen.uba.ar; 4 Institute of Biology, UNICAMP, Bertrand Russel s/n, 13083-865 Campinas-SP, Brazil; antonelif@yahoo.com; 5 Aquaculture Center of UNESP (CAUNESP), Prof. Paulo Donato Castellane s/n, 14884-900 Jaboticabal-SP, Brazil

**Keywords:** extracellular matrix, collagenase, protogynous diandric fish, germinal epithelium, gonad

## Abstract

Teleostei present great plasticity regarding sex change. During sex reversal, the whole gonad including the germinal epithelium undergoes significant changes, remodeling, and neoformation. However, there is no information on the changes that occur within the interstitial compartment. Considering the lack of information, especially on the role played by metalloproteinases (MMPs) in fish gonadal remodeling, the aim of this study was to evaluate the action of MMPs on gonads of sex reversed females of *Synbranchus marmoratus*, a fresh water protogynic diandric fish. Gonads were processed for light microscopy and blood samples were used for the determination of plasma sex steroid levels. During sex reversal, degeneration of the ovaries occurred and were gradually replaced by the germinal tissue of the male. The action of the MMPs induces significant changes in the interstitial compartment, allowing the reorganization of germinal epithelium. Leydig cells also showed an important role in female to male reversion. The gonadal transition coincides with changes in circulating sex steroid levels throughout sex reversion. The action of the MMPs, in the gonadal remodeling, especially on the basement membrane, is essential for the establishment of a new functional germinal epithelium.

## 1. Introduction

Sex change, one of the most controversial and remarkable expressions of plasticity in sexual development, can be observed in a number of teleost orders and families. Functional hermaphroditism was confirmed in 27 teleost families in seven orders [1]. A species or population is considered to exhibit functional hermaphroditism if a proportion of individuals function as both sexes at some time in an individual’s life history. This natural sex reversal process basically consists of the expression of both male and female reproductive functions in a single individual; the proliferation of secondary-sex gonadal tissue and the simultaneous degeneration of the primary-sex gonadal tissue [1,2,3,4].

There are two patterns recognized in fish sex reversal, simultaneous and sequential. Sequential hermaphroditism can vary in several ways, presenting as either protogynous or protandric. In cases of protogynous hermaphroditism, most frequently observed in teleosts, individuals first develop into females and later, have their functional ovaries gradually replaced by male tissue. Thus, a decrease in female gametogenesis and an increase in the male tissue activity are observed during sex reversal; then, protogynous species may show monandry, in which all males are secondary males arising from the sex reversed females, or diandry, in which there are two types of males: those that develop as primary males and those that are secondary males [1,5,6].

The swamp or marbled eel, *Synbranchus marmoratus* Bloch (1795), as *Monopterus albus* Zuiew (1793), the rice eel, also a Synbranchiformes [7], is a diandric protogynous fish that primarily—with an unrestricted lobular testis type—develops directly as males, while secondary males arise from the sex reversal of females [8,9,10,11,12]. They are widely distributed across Central and South America [13].

In this species, sex change is correlated to the length of the individual, where reversion takes place in those between 25.0 and 60.0 cm total length [9,12,14].

The sex reversal process in *Synbranchus marmoratus* begins in reproductive females and is characterized by a disorganization of the gonadal architecture; intense proliferation of myoid cells; appearance of new germline cysts located at the edges of the ovarian lamellae; massive degeneration of the female germ cells; intense phagocytic activity; increase of vascularization; and the presence of melanomacrophage centers [10,11,15].

During the gonadal development of teleosts, a constant remodeling of the interstitial tissue is required, according to the changes undergone by the germinal epithelium, either during reproductive cycles [16] or gonadal differentiation [17]. This remodeling of the connective tissue involves events of degradation and new synthesis of extracellular matrix components [18,19]. The degradation of the matrix components is effected by several proteolytic enzymes, with the matrix metalloproteinases (MMPs) being the main ones involved in this process [20].

Metalloproteinases (MMPs) are a group of structurally-related proteins (endopeptidases), calcium and zinc dependent, and active at physiological pH [21,22]. These enzymes act on the degradation of many components of the extracellular matrix during tissue remodeling and may be involved in the regulation of cell-cell and cell-matrix signaling [22,23]. In mammals, studies show that some of the major MMPs are involved in remodeling processes of the male germinal epithelium [24] and in the rupture of the ovarian follicle [25]. A recent study also shows the expression of proteinase genes and proteolytic enzymes in gonad development of the mouse [26]. However, in aspects regarding the reproductive biology in teleosts, there are few studies that relate gonadal remodeling to the action of the MMPs and none regarding their role during sex reversal.

Another important aspect during sex reversion is the role of the steroid hormones. In teleosts, sexual steroids are considered the main factor of gonadal sex development and reproduction and are involved on sex reversal in hermaphroditic species [1,3,12,15,27]. These hormones are produced by gonadal somatic cells: follicle and theca cells, in females; Sertoli and, especially, Leydig cells in males.

In most teleost fishes, as in other vertebrates, Leydig cells are usually located singly or in small clusters in the interstitial compartment of the testis [28]. Leydig cells have features of steroid-producing cells, such as steroid dehydrogenase enzymes (essential for the biosynthesis of most steroid hormones) and receptors for hormone/polypeptide growth factors [29,30]. In fact, different approaches and techniques have been used in teleost Leydig cells and have confirmed that these cells are the main source of testicular steroids [17,31,32,33,34,35].

Leydig cells, also in the swamp eel *Synbranchus marmoratus*, are known to have a steroidogenic function in both types of males [36]. Therefore, these cells may offer hormonal support to the development and proliferation of male structures during sex reversal. Consequently, in this protogynous hermaphroditic species, this cell type might be the earliest structure to arise in the ovary, thus considered the early transitional gonad.

Thus, in an attempt to determine the role of the matrix metalloproteinases (MMPs) in the gonadal tissue remodeling during sex reversal and examine whether Leydig cells are involved in the morphological and functional beginning of sex reversal in *Synbranchus marmoratus*, the transitional gonads were studied using histological high resolution light microscopy, polarizing light microscopy, and immunohistochemistry techniques for the detection of three types of metalloproteinases (MMP-2, MMP-9, and MMP-14) and the 3β-hydroxysteroid dehydrogenase (3β-HSD). Additionally, immunohistochemistry for the detection of proliferation of the Leydig cells and hormonal analysis of sex steroids were also performed.

## 2. Materials and Methods 

### 2.1. Animals

Adult *Synbranchus marmoratus* specimens, from 25 to 54.9 cm total length, were collected in the Tietê river, in the Penápolis region, São Paulo State, Southeastern of Brazil (21°17′ S; 49°47′ W). Fish were transferred to the laboratory and anesthetized by immersion in a solution of 0.1% benzocaine. Handling of animals was performed in compliance with international standards on animal welfare (Canadian Council on Animal Care, 2005—Ottawa, Canada), as well as being in compliance with the local Ethical Committee from Instituto de Biociências de Botucatu—Botucatu, SP—Brazil (n. 580-IBB—UNESP).

### 2.2. Sample Preparation for Light Microscopy

Animals were anesthetized and immediately following, were sacrificed by decapitation and the gonads were quickly removed. Samples were then fixed in glutaraldehyde 2% and paraformaldehyde 4% solution in Sorensen buffer (0.1 M at 7.2 pH) for at least 24 h at room temperature. After fixation, the samples were dehydrated in a crescent ethanol series and embedded in Historesin (Leica HistoResin^®^, Buffalo Grove, IL, USA). Cross sections (3 µm) were stained with Ferric Hematoxylin/Eosin (HE); Toluidine Blue (TB); Schiff Periodic Acid + Ferric Hematoxylin + Metanil Yellow (MY) [37]; and with the Reticulin Method that enhances basement membranes. The Reticulin stain [38] uses an oxidizing agent, potassium permanganate, to oxidize aldehyde groups. Subsequently, the oxidized aldehyde groups are detected by the deposition of positive silver ions followed by their reduction using formalin. The result is a black hue of the reticulin fibers. As reticulin fibers are part of basement membranes, the method clearly detects basement membranes.

The histological slides were also stained with Picrosirius Red and observed in polarized light for the localization of collagen fibers [39]. Gonadal tissues were evaluated by using a computerized image analyzer (Leica Qwin 2.5, Leica Microsystems, Buffalo Grove, IL, USA).

#### Immunohistochemistry for Metalloproteinases (MMPs), 3β-Hidroxysteroid Dehydrogenase(3β-HSD) Enzyme and Proliferating Cell Nuclear Antigen (PCNA)

For the detection of the MMPs (MMP14, MMP2, and MMP9), three β-HSD enzyme and PCNA samples were fixed in paraformoldehyde 4% for 1–3 h, embedded in Paraplast^®^ (Sigma-Aldrich, St. Louis, MO, USA) and sectioned at 5 µm. Sections were deparaffinized, hydrated in TBS buffer (Tris+phosphate buffer, 5 mM, pH 7.6), and then treated with 3% hydrogen peroxide for 15 min to quench endogenous peroxidase activity. Antigen retrieval was performed in a steam pan with citrate buffer (0.01 M; pH 6.0) for 20 min. After buffer rinse, slides were treated with a protein blocker (1% of non-fat powdered milk in TBS) for 15 min. They were subsequently incubated with the primary antibody, specific to each antigen: Anti-MMP14 (anti-rabbit, ab53712, ABCam), Anti-MMP2 (anti-rabbit, ab37150, ABCam), and Anti-MMP9 (anti-rabbit, ab38898, ABCam) polyclonal Ab. (1:50); anti-3-β HSD monoclonal Ab. (1:100) (anti-mouse, SC-100466, Santa Cruz, CA, USA); and Anti-PCNA monoclonal Ab. (1:300) (NCL-L-PCNA, clone PC10, Novocastra) for 2 h at room temperature in a moisture chamber. Next, slides were washed and incubated with MR HRP-Polymer (MACH4 Universal HRP Polymer Kit^®^) for 30 min. Immunostaining was visualized using 0.1% DAB (3′,3′-diamminobenzidine) in TBS buffer and 0.03% H_2_O_2_. Sections were lightly counterstained with Harris Hematoxylin, dehydrated, and mounted with DPX. Negative control sections were treated with TBS instead of primary antibody. Sections of ovaries from mouse specimens were used as the positive control.

### 2.3. Sex Steroid Levels

Blood samples of *Synbranchus marmoratus* were used for the quantification of plasma levels of steroids. Animals were anesthetized and peripheral blood was collected by puncture of the caudal vein with a heparin-coated needle, attached to a 3 mL syringe. Samples were centrifuged at 2500× *g* for 15 min at 4 °C to obtain plasma, which was stored at −20 °C.

The plasma profiles of 11-ketotestosterone (11-KT), testosterone (T), and 17β-estradiol (E2) were quantified by an enzyme-linked immunosorbent assay (ELISA) with commercial kits (for 11-KT: Cayman Chemical; for T and E2: Interkit). Minimum detectable concentrations in plasma samples were 45 pg mL^−1^ for E2, 57 pgmL^−1^ for 11-KT, and 59 pg mL^−1^ for T. The inter- and intra-assay coefficients of variation were 10.5% and 2.8 ± 0.2% for E2 (*n* = 8), 10.6% and 3.1 ± 0.2% for 11-KT (*n* = 8), and 9,6% and 4.4 ± 0.3% for T (*n* = 7), respectively. Main cross-reactivity (>1%; given by supplier) was detected with estradiol-3-glucoronide (17%) and estrone (4%) for the E2 antibody and with 5α-dihydrotestosterone (27.4%), 5β-dihydrotestosterone (18.9%), androstenedione (3.7%), and 11-KT (2.2%) for the T antibody. For 11-KT antiserum, cross-reactivity with other steroids was lower than 0.1%. Analyses were carried out following the manufacturer’s instructions, samples were assayed in duplicate, the standard curve was run for each ELISA plate, and the absorbance (450 nm for E2, 421 nm for 11-KT and T) measurements were performed in a microplate reader (Expert Plus ^®^, BIOCHROM, Cambridge, UK).

### 2.4. Statistical Analysis

Plasma levels of each hormone were grouped in distinct individual phases during the sex reversal of *Synbranchus marmoratus*. Values were expressed as mean ± SEM (standard error of the mean) and then subjected to variance analysis (one way ANOVA) and to a Tukey Test (Statistica*^®^* 7.0—StatSoft, Palo Alto, CA, USA). Means were considered statistically different at *p* < 0.05.

## 3. Results

### 3.1. Gonodal Structure of Synbranchus marmoratus

**Figure 1:**
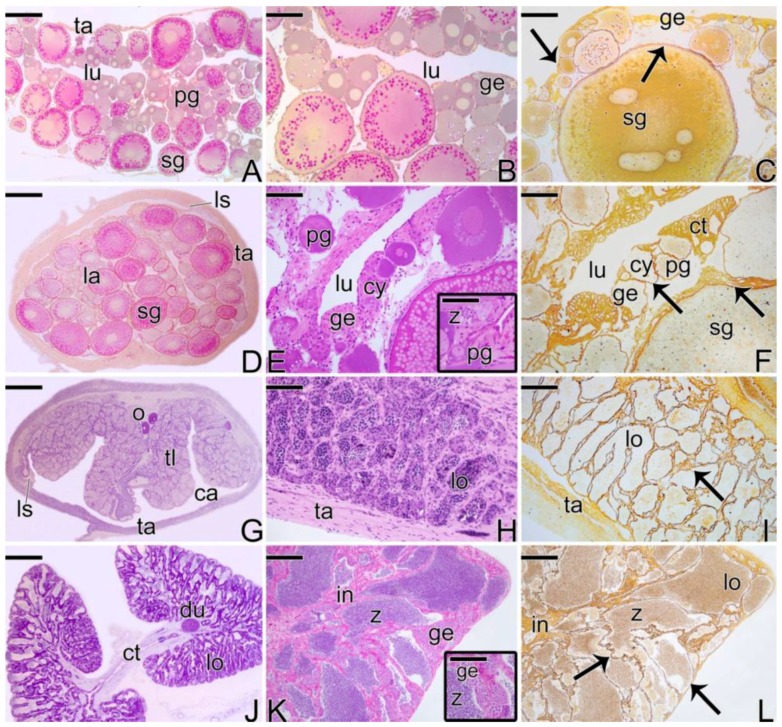
Four types of gonadal structures present in *S. marmoratus*: (**A**–**C**) Longitudinal section of ovary of a female. (**D**–**F**) Cross section of early transitional gonad, during the beginning of the sex reversal. (**G**–**I**) Cross section of testis of a secondary male. (**J**–**L**) Cross section of testis of a primary male. Note that the secondary male develops a single testis, whereas the primary male has a pair of testes. Note that the basement membrane, which supports the germinal epithelium, evidenced by the Reticulin Method, is present in all types of gonadal tissue organization (**C**,**F**,**I**,**L**). Tunica albuginea (ta), ovarian lumen (lu), primary growth oocyte (pg), secondary growth oocyte (sg), germinal epithelium (ge), lateral support (ls), ovigerous lamellae (la), germline cysts (cy), spermatozoa (z), connective tissue (ct), oocyte (o) testicular lamellae (tl), coelomic cavity (ca), testicular lobule (lo), testicular duct (du), interstitium (in), basement membrane (arrow). Staining: MY (**A**,**B**,**D**,**G**), HE (**E**,**H**,**J**,**K**), Reticulin Method (**C**,**F**,**I**,**L**). Bar: 300 µm (**A**,**D**,**G**,**J**), 50 µm (**B**,**C**,**E**,**E**-inset, **F**,**H**,**I**,**K**,**L**), 30 µm (K-inset).

*Synbranchus marmoratus* is a diandric protogynous hermaphrodite species, in which the population is composed of four types of sexual representatives (Figure 1): Females; Primary Males; Secondary Males; and Hermaphroditic Individuals. Primary males are those individuals that develop directly as male, while secondary males develop from a process of sex reversal of females, passing through a stage of intersexes.

The gonads are located dorsally to the digestive tract. They are elongated, cylindrical, and occupy 2/3 of the coelomic cavity, being connected to the urogenital papillae in the caudal portion. The female, male, or transitional gonads are attached to the dorsal wall of the coelomic cavity by a connective tissue membrane—the mesentery.

The ovaries (Figure 1A) constitute a single saculiform organ, surrounded by a thick capsule of connective tissue (the tunica albuginea). It presents a lumen, delimited by a germinal epithelium, which borders the ovigerous lamellae (Figure 1B). The germinal epithelium, supported by a basement membrane (Figure 1C), is composed of somatic and germ cells. In cross sections, ovigerous lamellae are projected into the organ (ovarian cavity) from the ventral region, being attached to the tunica albuginea by lateral supports.

Initially, the female gonads that undergo the sex change process (Figure 1D) have the same characteristics as an ovary, as described above. At the beginning of the transitional process, the epithelium becomes composed of a larger number of somatic and germ cells. The connective tissue (Figure 1E) becomes more developed. Thus, the germinal epithelium still supported by a basement membrane (Figure 1F) presents a greater number of germline cysts immersed in a connective tissue and surrounded by a basement membrane, which separates them from the interstitial compartment.

After the reversion, the “new” male gonad (Figure 1G), named secondary males, is drafted on a female gonad. Thus, the testis of the secondary male becomes a single structure, externally delimited by a connective tissue capsule (the former tunica albuginea of the ovary) (Figure 1G). The new testicular lobules, called testicular lamellae, develop on the former ovigerous lamellae, showing the same aspects as a primary male testes, that is, they are formed by a germinal epithelium supported by a basement membrane (Figure 1H,I). However, unlike primary males, testes that come from sex reversal have lateral supports (Figure 1G), which attach the old lamellae to the capsule of connective tissue. In addition, the lumen is maintained during the process of sex reversal. Thus, the testes of the secondary male present a non-functional coelomic cavity (pseudo-ovarian cavity) (Figure 1G).

The testes of primary males are paired organs, joined medially by connective tissue (Figure 1J). Histologically, they present a lobular unrestricted type organization (Figure 1K). The germinal epithelium of the testicular lobules, supported by a basement membrane (Figure 1L), is composed of somatic and male germ cells.

### 3.2. Female Gonad of Synbranchus marmoratus

**Figure 2:**
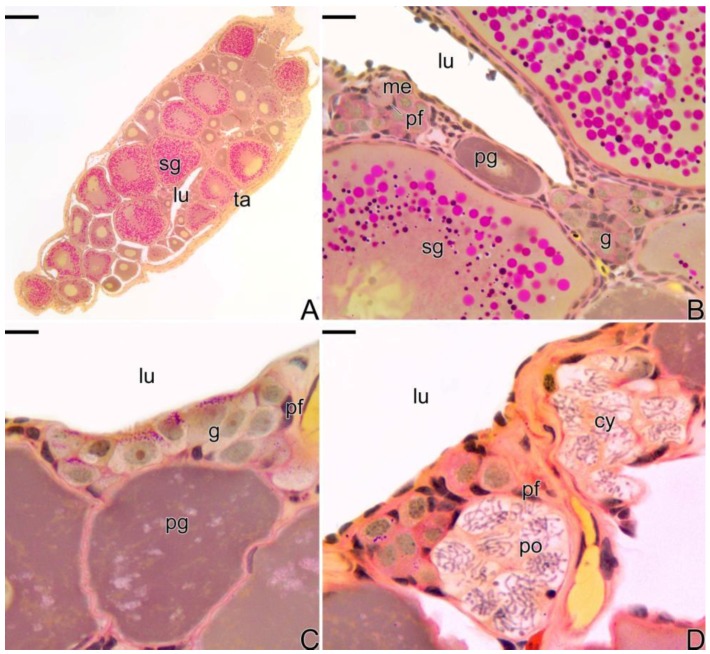
Histological section of ovary of *S. marmoratus.* (**A**) Cross section of ovary. (**B**–**D**) Details of A, showing the germinal epithelium, with germline cysts. Secondary growth oocyte (sg), ovarian lumen (lu), tunica albuginea (ta), metaphase (me), primary growth oocyte (pg), oogonia (g), pre-follicle cell (pf), pachytene oocyte (po), germline cyst (cy). Staining: MY. Bar: 200 µm (**A**), 20 µm (**B**), 10 µm (**C**,**D**).

As was described above, the ovaries of *Synbranchus marmoratus* present an ovarian cavity in which ovigerous lamellae are projected (Figure 2A). Along the ovigerous lamellae is the female germinal epithelium (Figure 2B), constituted by squamous somatic cells, with a basophilic nucleus andoogonia. These are located throughout the germinal epithelium, isolated or forming cell clusters, resulting from cell proliferation. Oogonia are surrounded by somatic cells forming germline cysts. In the cysts, the oogonia divide by mitosis and through the oogenesis process, enter into meiosis, giving rise to oocytes. In these niches of cell proliferation and differentiation, the formation of ovarian follicles occurs in a permanently active epithelium (Figure 2B–D).

### 3.3. The Gonadal Remodeling during the Sex Reversal—Formation of the Gonad of the Secondary Male

#### 3.3.1. The Female Early Transitional Gonadal Tissue

The process of sexual reversion in *Synbranchus marmoratus* begins in a functional ovary, from the border of the ovarian cavity. This ovary shows an active female germinal epithelium, formed by oogonia, prophase oocytes, and primary and secondary growth oocytes.

**Figure 3:**
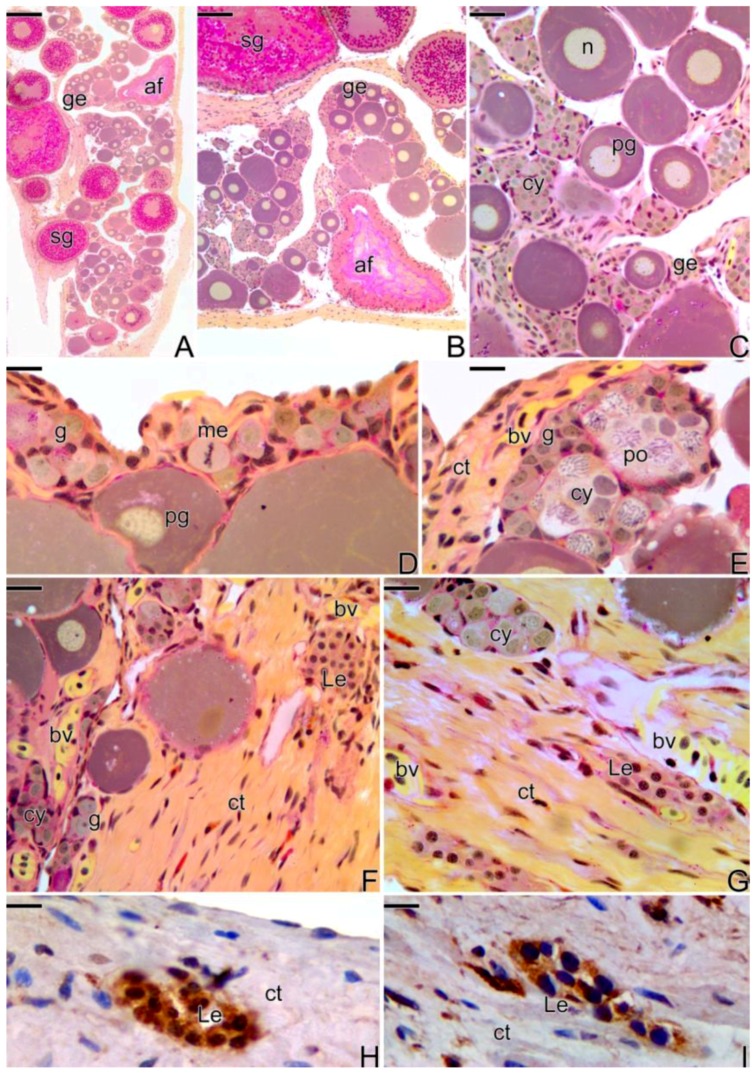
Histological sections of ovary at the beginning of gonadal transition of *S. marmoratus*. Note the presence of atretic follicles (**A**,**B**), a greater number of germline cysts, along the epithelium (**C**–**E**), increased blood vessels and connective tissue in the interstitial compartment, and the presence of Leydig cells, close to the blood vessels (**F**–**H**) Immunohistochemistry for detection of PCNA. The labeled cells that show positive response to PCNA (**H**) are Leydig cells. (**I**) Immunohistochemistry for detection of 3β-HSD enzyme, indicating that it is a steroidogenic cell. Germinal epithelium (ge), secondary growth oocyte (sg), atretic follicle (af), germline cyst (cy), nucleus (n), primary growth oocyte (pg), metaphase (me), gonia (g), connective tissue (ct), pachytene oocyte (po), blood vessel (bv), Leydig cell (Le). Staining: MY (**A**–**G**). Counterstaining: Harrys Hematoxylin (**H**,**I**). Bar: 200 µm (**A**), 100 µm (**B**), 20 µm (**C**,**F**), 10 µm (**D**–**I**).

The first signs that indicate the beginning of the transition are a structural disorganization of the female gonad, mainly consisting of the entrance into atresia of some secondary growth oocytes (Figure 3A,B). Concomitantly, there is a thickening of the connective tissue, adjacent to the epithelium (Figure 3C–F) that borders the ovigerous lamellae (Figure 3B,C). As a consequence of this thickening, there is the formation of invaginations from the coemolic cavity towards the epithelium (Figure 3D). In this region, a proliferation of gonia (Figure 3D) starts: entering into meiosis and increasing the number of cysts in the germinal epithelium (Figure 3E). Thus, the male gonadal tissue, initially scarce, gradually replaces the female germinal epithelium that initiates a constant regression. Just below the basement membrane, an increase of the collagen fibers occurs in the underlying interstitial tissue (Figure 3E,F).

Throughout the process of folliculogenesis and entrance of the oocyte into primary growth, the prophase oocytes, as well as the primary growth oocytes (Figure 3A–F), remain involved by somatic cells, as well as the pre-follicle and follicle cells, respectively, which synthesize a basement membrane, segregating them from the interstitial components. The basement membrane often becomes tortuous, next to the interstitial compartment, remaining rectilinear around the ovarian follicles.

At this stage of the sex reversal process, a proliferation of Leydig cells (Figure 3F,G) was observed in the interstitial tissue, which showed a positive response to immunohistochemistry for PCNA (Figure 3H) and for the detection of 3β-HSD (Figure 3I). Leydig cells have an oval shape and a small spherical nucleus with compacted chromatin (Figure 3F). They usually form cell clusters, either in the interstitial tissue or in the connective tissue capsule, often close to the blood vessels (Figure3G).

#### 3.3.2. The Female Intermediary Transitional Gonad Tissue

**Figure 4:**
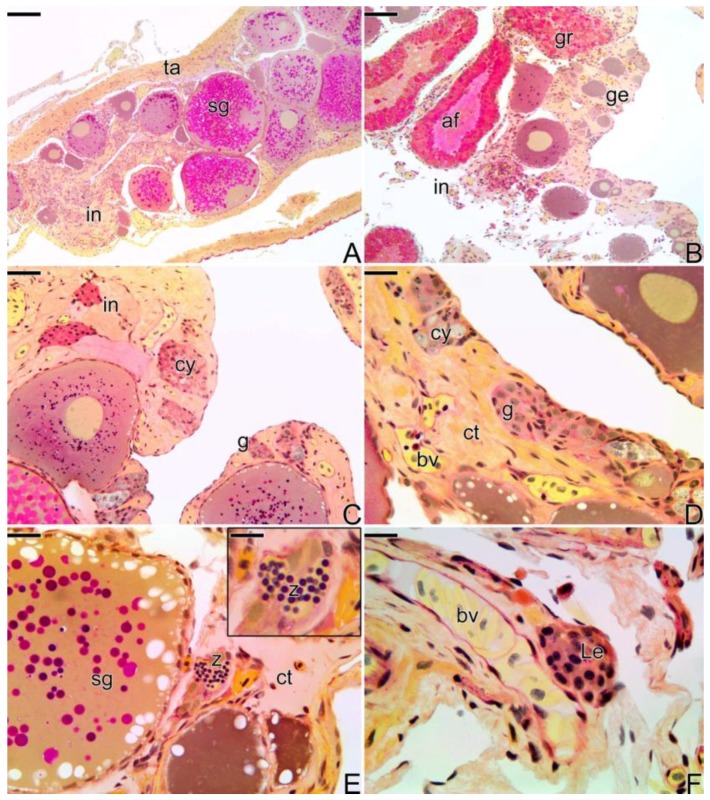
Histological sections of intermediary transitional gonad (ovary) of *S. marmoratus*. (**A**) Ovary with primary and secondary growth oocytes. (**B**) Atretic follicle and granulocytes in large quantity in gonadal tissue. (**C**,**D**) Male germinal epithelium developing gradually. (**E**) First spermatozoa produced. E-inset) Detail of the spermatozoa. (**F**) Leydig cells near blood vessels. Tunica albuginea (ta), interstitium (in), granulocyte (gr), secondary growth oocyte (sg), germinal epithelium (ge), atretic follicle (af), germline cyst (cy), connective tissue (ct), gonia (g), blood vessel (bv), Leydig cell (Le), spermatozoa (z). Staining: MY. Bar: 200 µm (**A**), 100 µm (**B**), 20 µm (**C**–**E**), 10 µm (**E**-inset, **F**).

As the gonadal remodeling progresses, increasing the structural disorganization of the gonad (Figure 4A), atretic follicles are frequently observed (Figure 4B), while the female portion of the gonad remains located in the peripheral region of the gonadal tissue, opposite to the germinal epithelium, which is being invaded by male gonadal tissue (Figure 4C,D). Hyperplasia of the connective tissue of the capsule (Figure 4A) and a strongly increased amount of blood vessels can be observed, both in the center of the gonad and in the region underlying the epithelium (Figure 4C,D).

At this stage, it is possible to observe the first spermatozoa in the lumen of small intra-tissue spaces newly formed (Figure 4E). These spaces constitute the primordium of the first testicular lobules, which will be established at the end of the process of sex reversion. The interstitial tissue develops progressively, being constituted by a large amount of fibroblasts, collagen fibers, myoid cells, and granulocytes. Near the blood vessels, the presence of groups of Leydig cells is often noted (Figure 4F).

#### 3.3.3. The Final Transitional Gonad—The Intersex

**Figure 5:**
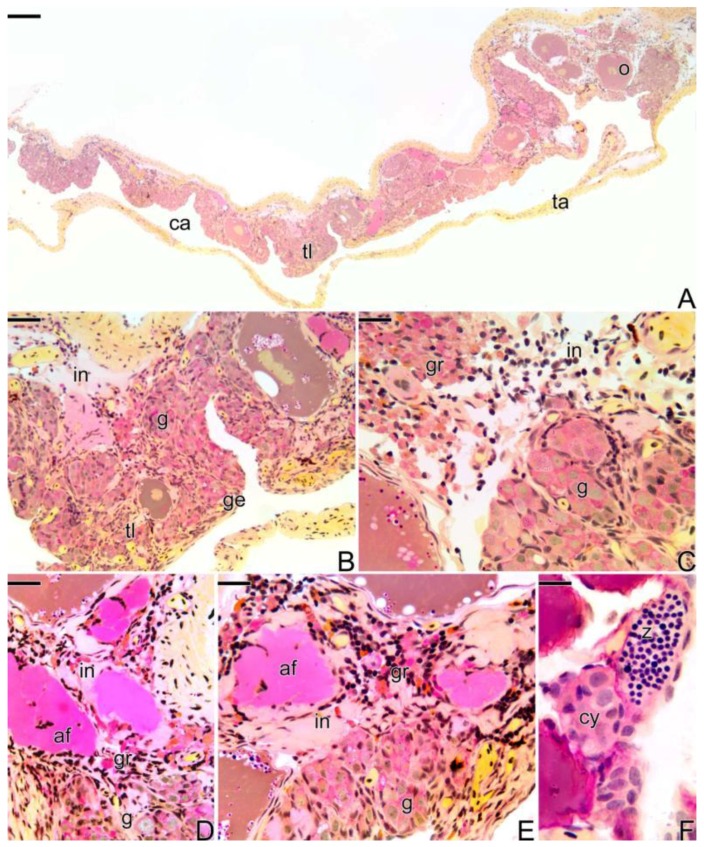
Histological section of final transitional gonad—the intersex of *S. marmoratus*. (**A**) General view of the gonad. (**B**–**F**) Details of A. Note the organization of the gonad in testicular lamellae (**B**) and the large amount of granulocytes that invade the interstitial tissue (**C**–**E**) close to atretic follicles (**D**,**E**). (**F**) Presence of the first spermatozoa. Coelomic cavity (ca), testicular lamellae (tl), tunica albuginea (ta), oocyte (o), interstitium (in), germinal epithelium (ge), gonia (g), granulocytes (gr), atretic follicle (af), spermatozoa (z). Staining: MY. Bar: 100 µm (**A**), 50 µm (**B**), 20 µm (**C**–**E**), 10 µm (**F**).

At the final stage of sex reversal, the gonad becomes less thick due to the degeneration of the female gonadal tissue and predominance of the male gonadal tissue (Figure 5A). It is possible to observe some remaining oocytes in primary and secondary growth, but the male germline cysts occupy most of the gonad (Figure 5A–C). The distribution of the male gonadal tissue on the old ovigerous lamellae is observed (Figure 5A,B). The male gonadal tissue is formed by cysts containing spermatogonia and spermatocytes (Figure 5B,C). These are organized in cell clusters, such as acinar structures, in which there is not a fully defined lumen yet, except in some regions where spermatozoa are present in the lumen of a rudimentary testicular lobule (Figure 5F). Granulocytes infiltrate in large amounts within the interstitial tissue, being quite frequent near the atretic follicles and melanomacrophage centers (Figure 5D,E; Supplementary Figure S1).

#### 3.3.4. The Gonadal Tissue of the Secondary Male

**Figure 6:**
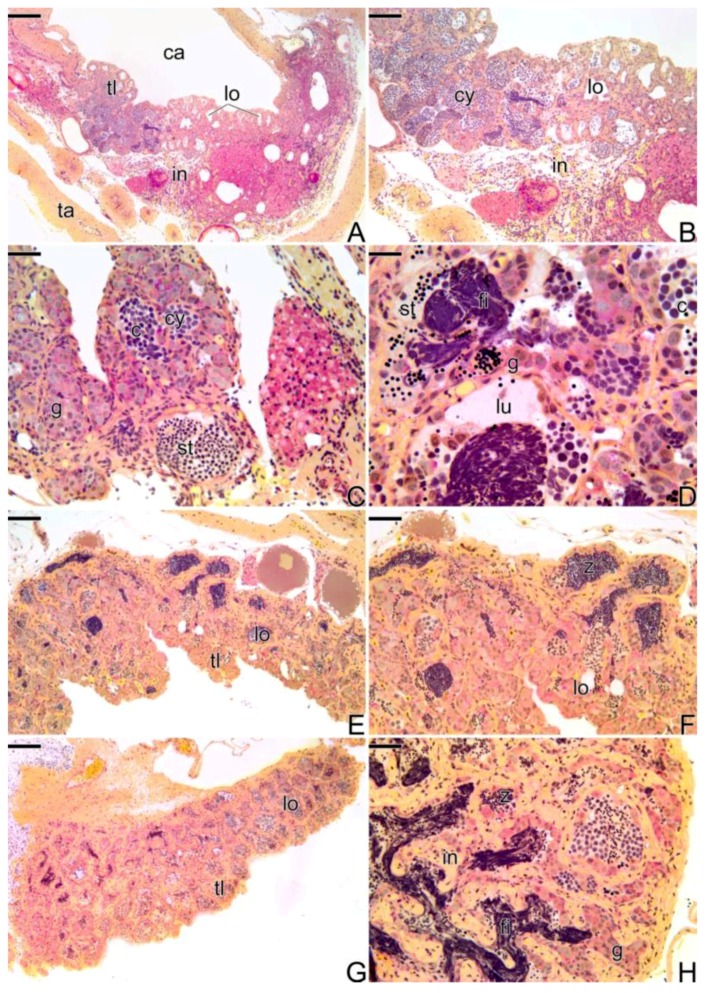
Histological sections of testes of secondary males of *S. marmoratus*. Note the progression of spermatogenesis, throughout the process of testicular development. Coelomic cavity (ca), interstitium (in), tunica albuginea (ta), testicular lamellae (tl), testicular lobules (lo), gemline cysts (cy), spermatocytes (c), spermatogonia (g), spermatids (st), basophilic filaments (fi), testicular lumen (lu). Staining: MY. Bar: 200 µm (**A**,**E**,**G**), 100 µm (**B**), 20 µm (**C**), 15 µm (**D**), 30 µm (**F**).

At the end of the sex reversal process, the gonad does not presents intersex characteristics, showing predominantly male elements, such as the presence of testicular lobules (Figure 6A,B). The interstitial tissue underneath the male germinal epithelium is still disarranged. It is possible to observe some areas of necrosis with eosinophilic cells and a few remaining oocytes. The testicular lobules are formed from the acinar structures, constituted by germ cells. These germ cells, the spermatogonia, once encysted by the Sertoli cells, move away from one another, in the same cluster, forming small testicular lobules (Figure 6B). With the proliferation of the spermatogonia, the lobules grow, presenting a larger extension and a wider testicular lumen. Thus, the gonadal tissue is now completely remodeled into a male gonad, with male germinal epithelium completely established and identical to the germinal epithelium of the testis of a primary male. Anatomically, the testis of the secondary male remains quite similar to the anatomical structure of the ovary.

In the germinal epithelium of the lobules, the spermatogenesis begins and it is possible to observe all types of male germ cells (spermatogonia, spermatocytes, and spermatids) (Figure 6C,D). The germinal compartment, supported by a basement membrane, is composed of Sertoli and germ cells that give rise to sperm. At the end of the spermiogenesis, the lumen of the testicular lobules gradually becomes fully filled by spermatozoa (Figure 6E,F). It is still possible to observe a few remaining oocytes (Figure 6E).

Spermatogonia and pre-Sertoli cells present the same structural characteristics of oogonia and pre-follicle cells. The pre-Sertoli cells have a triangular nucleus, sparse cytoplasm, and cytoplasmic projections that interpose gradually between spermatogonia. Thus, spermatogonia are gradually and individually affected by cytoplasmic expansions of the now Sertoli cells, forming cysts delimited by cytoplasmic extensions of the Sertoli cells.

Now, the testis is fully formed and the secondary male is able to reproduce (Figure 6G,H). During the process, it is common to observe the presence of basophilic filaments distributed throughout the gonadal tissue (Figure 6H).

#### 3.3.5. The Male Gonad of *Synbranchus marmoratus*—Primary Male

**Figure 7:**
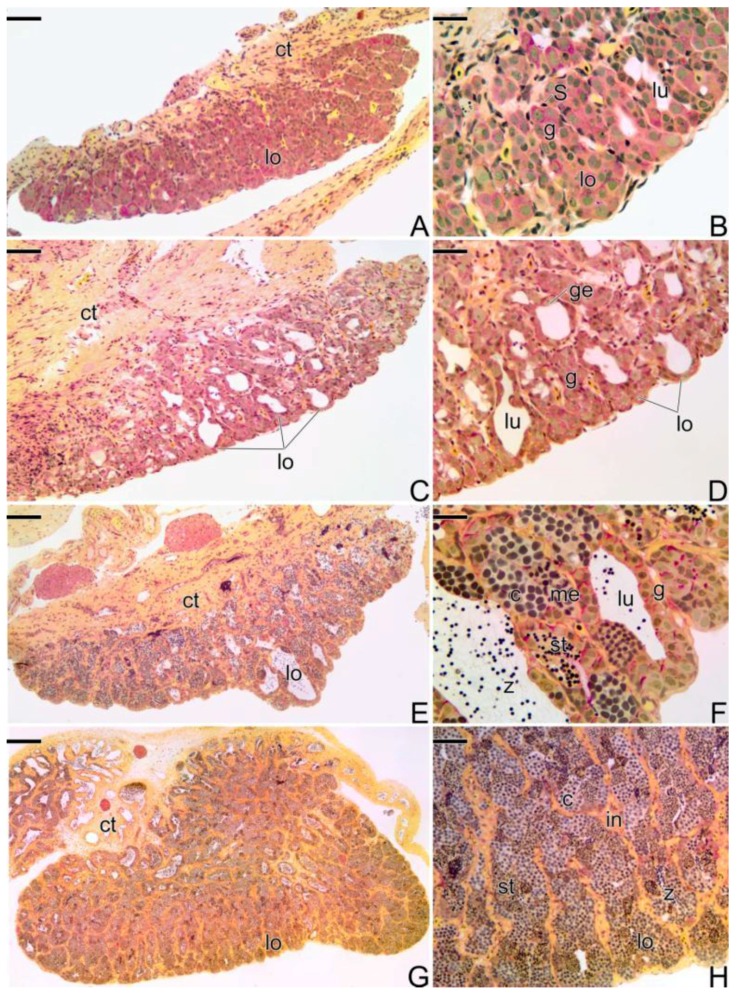
Histological sections of testes of primary males of *S. marmoratus*. Note the development of the testicular lobules, according to the progress of hte spermatogenesis. (**A**,**B**) Early testicular development. Germinal epithelium ir formed by Sertoli cells and spermatogonia. (**C**,**D**) Testicular lobules expand, increasing the testicular lumen. (**E**,**F**) Beginning of the spermatogenesis. (**G**,**H**) Testicular lobules are filled by spermatozoa. Connective tissue (ct), testicular lobules (lo), spermatogonia (g), Sertoli cell (S), testicular lumen (lu), germinal epithelium (ge), spermatocytes (c), metaphase (me), spermatids (st), spermatozoa (z). Staining: MY. Bar: 100 µm (**A**,**C**,**E**), 20 µm (**B**,**D**,**F**), 200 µm (**G**), 50 µm (**H**).

During all reproductive phases and/or the period of testicular differentiation of the primary male of *Synbranchus marmoratus*, the gonads remain paired, elongated, and cylindrical organs. At the beginning of the development, the gonadal tissue presents small testicular lobules, formed only by a germinal epithelium composed of cysts of spermatogonia, with frequent mitotic divisions. The luminal compartment of the lobes is still reduced and empty (Figure 7A,B).

Gradually, the spermatogonia continue to proliferate, enlarging the luminal compartment (Figure 7C,D). Spermatogonia enter into meiosis, initiating the spermatogenesis, which becomes continuous. With the increase of germ and somatic cells, the testis expands in width and length (Figure 7E). Spermatocytes in the early stages of the meiotic prophase become numerous in the testis (Figure 7F). Spermatozoa begin to be produced (Figure 7F).

With the progress of spermatogenesis, cysts of spermatid become numerous. As a consequence of the increase of germline cysts, there is an increase in the length of the lobules and the testis expands (Figure 7G). Cysts of spermatogonia are distributed throughout the testicular lobule among cysts of other germ cells, such as spermatocytes and spermatids, characterizing the testicular organization as an unrestricted lobular type (Figure 7H). The adjacent testicular lobules are separated from each other by highly developed interstitial tissue (Figure 7H). The production of spermatozoa increases and the testicular lumen, now more extensive, is filled by a large number of these cells (Figure 7H).

### 3.4. Detection of the Matrix Metalloproteinases MMP-9, MMP-2 and MMP-14 during the Sex Reversal

**Figure 8:**
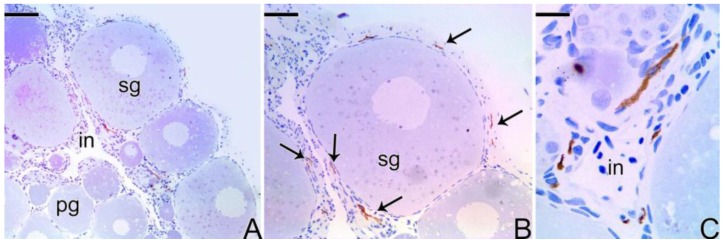
Immunohistochemistry for detection of MMP-9 metalloproteinase in ovary of *S. marmoratus*. Note the positive labeling in the interstitial cells (arrow). Primary growth oocyte (pg), secondary growth oocyte (sg), interstitium (in). Counterstaining: Harrys Hematoxylin. Bar: 80 μm (**A**), 30 μm (**B**), 15 μm (**C**).

In females of *Synbranchus marmoratus*, the MMP-9 enzyme was detected through immunohistochemistry techniques in a few ovarian regions, especially in cells of the ovarian stroma and in some regions of the connective tissue capsule (tunica albuginea) (Figure 8). MMP-2 and MMP-14 were not detected in this ovarian stage.

**Figure 9:**
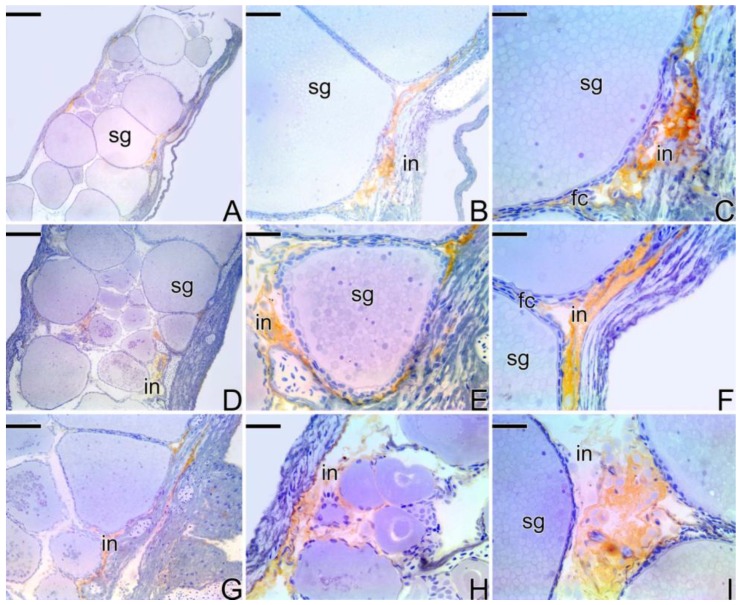
Immunohistochemistry for detection of MMP-14 (**A**–**C**), MMP-2 (**D**–**F**), and MMP-9 (**G**–**I**) metalloproteinases in an early transitional ovary of *S. marmoratus*. Secondary growth oocyte (sg), interstitium (in), follicle complex of the oocyte (fc). Counterstaining: Harrys Hematoxylin. Bar: 50 µm (**A**), 30 µm (**B**,**H**), 15 µm (**C**,**E**,**F**,**I**), 40 µm (**D**,**G**).

During the beginning of the transitional process of the gonad, the labeling for the three types of metalloproteinases (MMPs) becomes intense and the immunolocalization coincides in all cases (Figure 9), predominating in ovarian stromal cells and around the primary and secondary growth oocytes, in the theca cells. Some mesenchymal cells were positively labeled for MMP-9 (Figure 9H).

**Figure 10:**
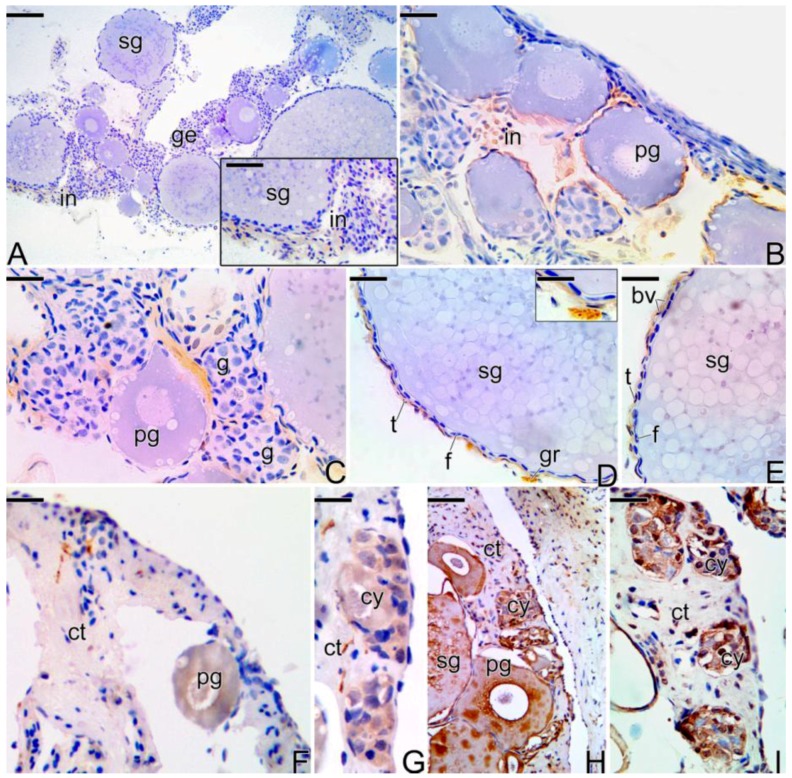
(**A**–**I**) Immunohistochemistry for detection of MMP-2 (**A**), MMP-9 (**B**–**D**), and MMP-14 (**E**) metalloproteinases in the intermediate transitional ovary of *S. marmoratus*. (**F**–**I**) Immunohistochemistry for detection of MMP-14 (**F**–**G**), MMP-9 (**H**), and MMP-2 (**I**) metalloproteinases in final transitional gonad (intersex) of *S. marmoratus*. Secondary growth oocyte (sg), interstitium (in), germinal epithelium (ge), primary growth oocyte (pg), gonia (g), granulocyte (gr), theca cell (t), follicle cells (f), germline cyst (cy), connective tissue (ct). Counterstaining: Harrys Hematoxylin (**A**–**I**). Bar: 100 µm (**A**), 40 µm (**A**-inset), 20 µm (**B**–**I**), 15 µm (**G**), 25 µm (**H**), 5 µm (**D**-inset).

During the intermediate transitional stage (Figure 10A–E), the gonad shows interstitial cells marked positively at the detection of MMP-2, but at a lower intensity compared to the previous stage (Figure 10A). Some gonia, theca cells, ovarian stromal cells, and granulocytes also showed a positive response to MMP-9 (Figure 10B–D). Also at this stage, the MMP-14 enzyme was detected only in theca cells (Figure 10E).

During the intersex phase (Figure 10F–I), MMP-14 was poorly detected in some regions of connective tissue, as well as in primary growing oocytes and germline cysts of spermatogonia (Figure 10F,G). MMP-9 was detected in primary and secondary growth oocytes, as well as in germline cysts, showing a more intense marking when compared to the MMP-14 (Figure 10H). An intense marking of MMP-2 was also found in germline cysts (Figure 10I).

**Figure 11:**
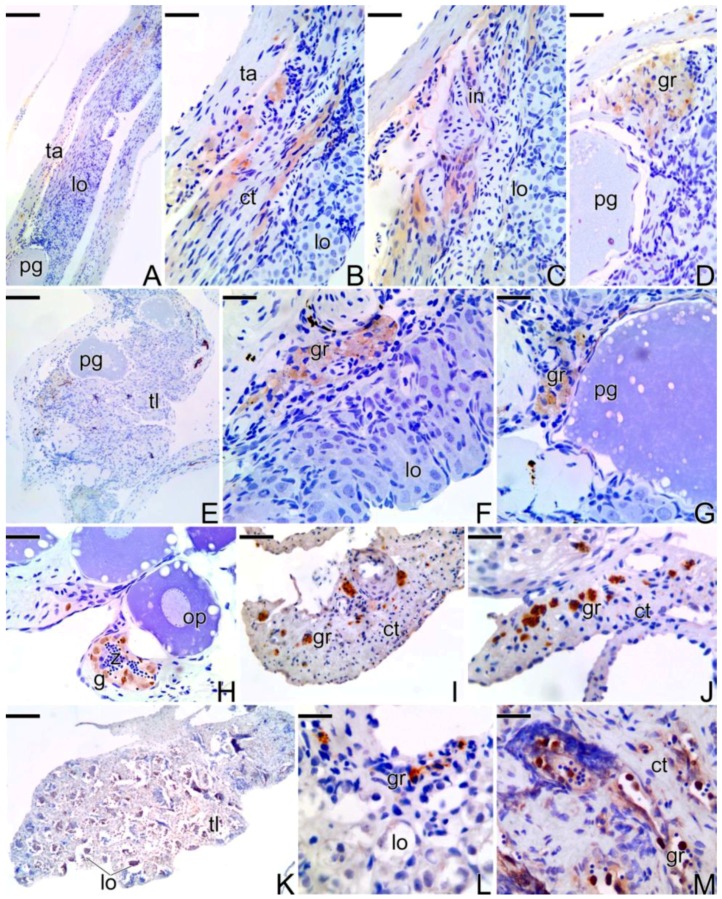
Immunohistochemistry for detection of MMP-9 (**A**–**D**,**J**), MMP-2 (**E**,**F**,**I**,**K**–**M**), and MMP-14 (**G**) metalloproteinases in testis of secondary males of *S. marmoratus*. Note the decline in the expression of the MMPs according to the end of the sex reversal process. Tunica albuginea (ta), testicular lobules (lo), connective tissue (ct), interstitium (in), primary growth oocyte (pg), granulocytes (gr), spermatogonia (g), testicular lamellae (tl), spermatozoa (z). Counterstaining: Harrys Hematoxylin. Bar: 80 µm (**A**,**E**), 40 µm (**B**,**C**,**I**), 30 µm (**D**,**F**,**G**), 20 µm (**H**,**J**,**L**,**M**).

At the end of the sex reversal process, the gonad of the secondary male shows a weak reaction to the MMP-2, MMP-9, and MMP-14 proteins. MMP-9 was detected in somatic cells, in the dorsal region of the gonad, as well as in interstitial cells (Figure 11A–C). Granulocytes present in melanomacrophage centers also showed a positive response to MMP-9 detection, being absent in the cytoplasm of oocytes or in their follicle complexes (Figure 11D). The MMP-2 enzyme was not detected in the majority of the germline cysts (Figure 11E), but it showed a positive response in granulocytes located in the melanomacrophage centers (Figure 11F) and in some spermatogonia located in lobules full with sperm (Figure 11H). MMP-14 was expressed in granulocytes and in theca cells of follicle complexes (Figure 11G).

After the beginning of the spermatogenesis, the testis of the secondary male (Figure 11K) shows a positive response to MMP-2 (Figure 11I) and MMP-9 (Figure 11J) in granulocytes from the connective tissue capsule. Granulocytes present in the interstitial compartment of the testis (Figure 11L) also show a positive response to MMP-2, which is also detected in interstitial tissue cells and granulocytes present inside the lumen of testes (Figure 11M).

**Figure 12:**
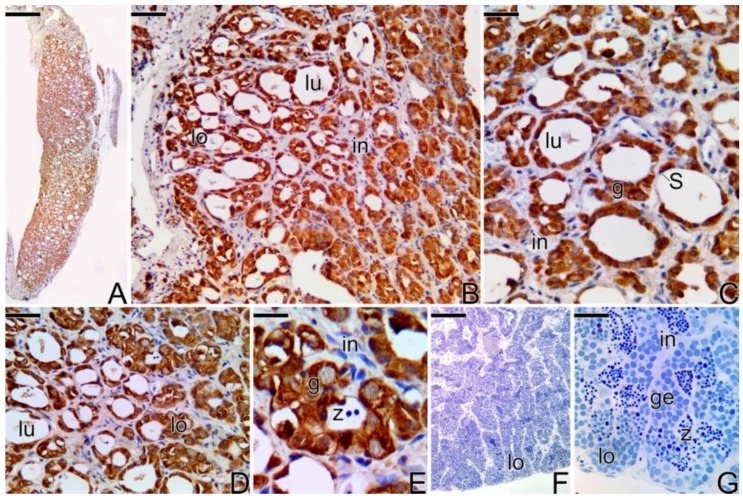
Immunohistochemistry for detection of metalloproteinases in testis of primary males of *S. marmoratus*. (**A**) General view of the testis. (**A**–**E**) Immunohistochemistry for detection of MMP-2 (**A**,**B**), MMP-9 (**C**), and MMP-14 (**D**,**E**). (**F**,**G**) In totally developed testes, there was no positive response to any of the MMPs. Testicular lobules (lo), testicular lumen (lu), interstitium (in), Sertoli cell (S), spermatogonia (g), spermatozoa (z), germinal epithelium (ge). Counterstaining: Harrys Hematoxylin. Bar: 100 µm (**A**,**F**), 40 µm (**B**,**D**), 20 µm (**C**), 10 µm (**E**), 30 µm (**G**).

In testes of the primary males, MMP-2 and MMP-9 (Figure 12C) were detected in the spermatogonia that constitute the germinal epithelium of the testicular lobules. The same positive result was observed for MMP-14 (Figure 12D,E). After the beginning of the spermatogenesis, none of the enzymes was detected, not even in granulocytes or in melanomacrophage centers (Figure 12F,G).

The expression of the metalloproteinases (MMP-2, MMP-9 and MMP-14) was also confirmed by the negative control sections of the *Synbranchus marmoratus* treated with TBS instead of primary antibody (Supplementary Figure S2) and by the positive control in sections of ovaries from mouse specimens (Supplementary Figure S3).

The activity of the gelatinases (MMP-2 and MMP-9) was confirmed by in situ zymography in all types of gonads analyzed: ovary (Supplementary Figure S4A-H), transitional ovary during intersex (Supplementary Figure S4I-P), testis of secondary male (Supplementary Figure S5A-H) and testis of primary male (Supplementary Figure S5I-P).

### 3.5. Remodelation of the Collagen Fibers in Synbranchus marmoratus

Through the Picrosirius Red staining, a differential distribution of the collagen fibers was observed during the gonadal sex reversal of *Synbranchus marmoratus*.

**Figure 13:**
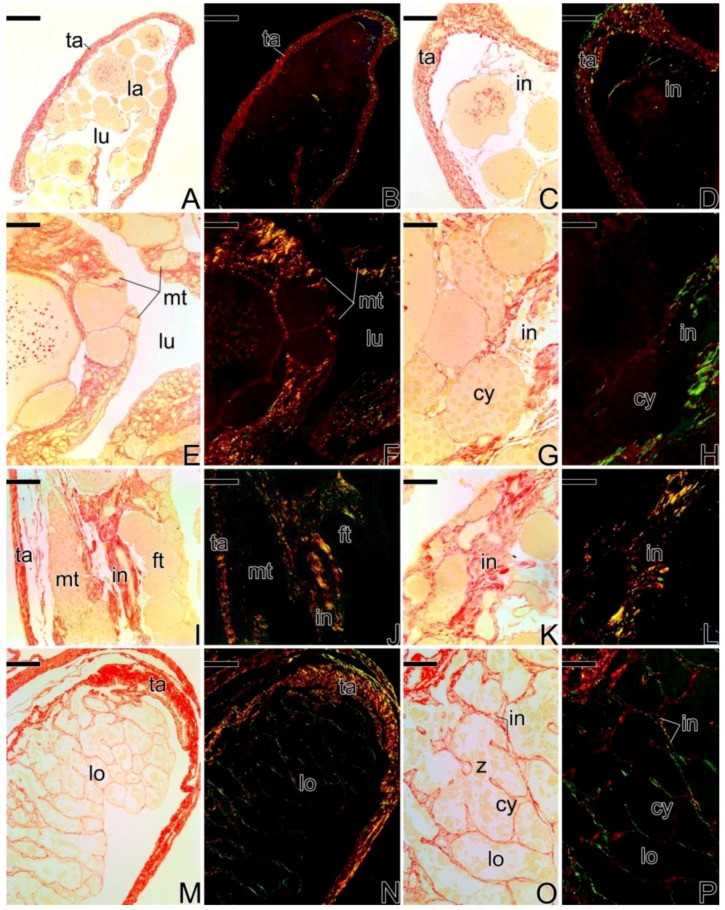
Histological sections of gonads of *S. marmoratus* showing gonadal remodeling during sex reversal. Picrosirius Red staining. (**A**,**C**,**E**,**G**,**I**,**K**,**M**,**O**) unpolarized light. (**B**,**D**,**F**,**H**,**J**,**L**,**N**,**P**) polarized light. (**A**–**D**). Ovary. Note the collagen fibers in the tunica albuginea are highly birefringent, while those of the interstitium are less birefringent. (**E**–**H**) Early transitional gonad. The collagen fibers are predominant next to the germinal epithelium, in that the remodeling begins. (**I**–**L**) Final transitional gonad. The birefringent collagen fibers are now found in the interstitium, in the central region of the gonad. (**M**–**P**) Testis of secondary male, showing the tunica albuginea defined as in the female. In the interstitial compartment, the organized collagen fibers show the definitive establishment of the germinal and interstitial compartment. Tunica albuginea (ta), ovigerous lamellae (la), ovarian lumen (lu), interstitium (in), male gonadal tissue (mt), female gonadal tissue (ft), testicular lobule (lo), germline cysts (cy), spermatozoa (z). Staining: Sirius red (red) and Picric Acid (yellow). Bar: 300 µm (**A**,**B**), 200 μm (**B**,**C**,**I**,**J**,**M**,**N**), 100 μm (**E**,**F**), 40 µm (**G**,**H**,**K**,**L**,**O**,**P**), 250 µm (**M**,**N**).

In the females, the collagen fibers of the tunica albuginea are strongly birefringent, presenting reddish coloration (Figure 13A–D), corresponding to type I collagen.

During the sex reversal, birefringent collagen fibers especially were observed next to the germinal epithelium that begins its remodeling. These fibers present yellowish and greenish tones corresponding to younger and older collagen fibers, respectively (Figure 13E–H). A similar pattern was observed at the end of the gonadal transition process (Figure 13I–L). In these transitional stages, there is a predominance of collagen fibers adjacent to the epithelium. However, the fibers within the gonadal tissue show low or no birefringence, such as in interstitial areas around oocytes or underlying the newly formed testicular lobules (Figure 13E–L).

In gonads of secondary male specimens, the tunica albuginea presents thicker collagen fibers than in the earlier transitional stages, with the prevalence of more mature collagen (Figure 13M,N). In this stage, there is a high birefringence of the less mature collagen (in green) around the testicular lobules, corresponding to the components of the basement membrane (Figure 13M–P).

**Figure 14:**
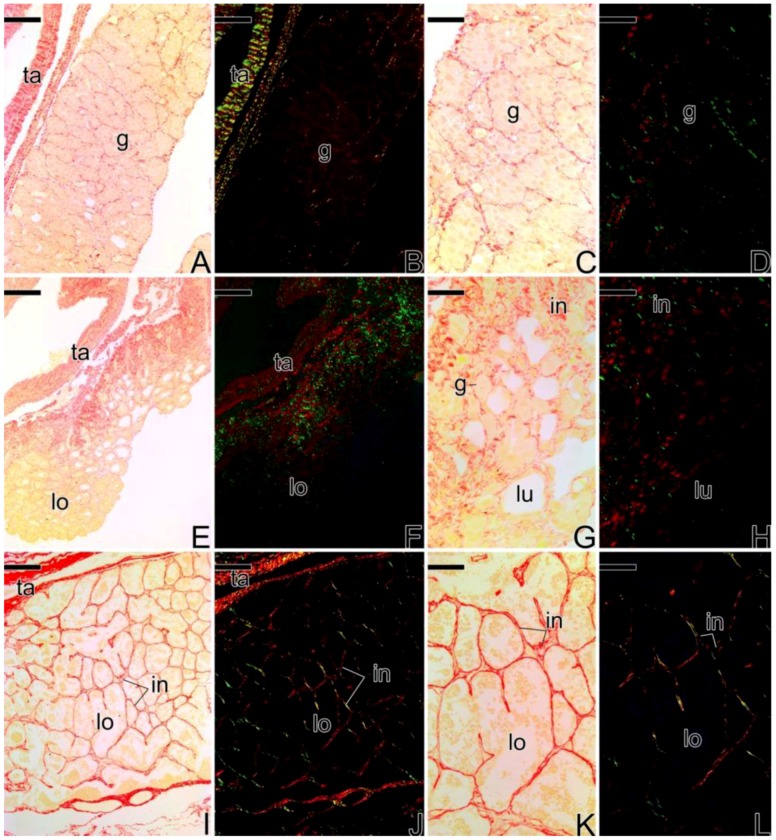
Histological sections of the stages of testicular development of primary males of *S. marmoratus*. Picrosirius Red staining. (**A**,**C**,**E**,**G**,**I**,**K**) unpolarized light. (**B**,**D**,**F**,**H**,**J**,**L**) polarized light. (**A**–**D**) Testis at the beginning of the development, with germinal epithelium formed only by spermatogonia. (**E**–**H**) Testis with germinal epithelium formed by cysts of spermatogonia, spermatocytes, and spermatids. There is no production of spermatozoa yet. The collagen fibers of the interstitium are more intense than the previous stage. (**I**–**L**) Testis of primary male capable of reproducing, showing the tunica albuginea defined. In the interstitial compartment, the collagen fibers are quite organized and defined. Tunica albuginea (ta), spermatogonia (g), interstitium (in), testicular lumen (lu), testicular lobule (lo). Staining: Sirius red (red) and Picric Acid (yellow). Bar: 100 µm (**A**,**B**,**I**,**J**), 50 μm (**C**,**D**,**G**,**H**,**K**,**L**), 150 μm (**E**,**F**).

During the development of of primary male testes (Figure 14), the collagen fibers of the tunica albuginea show greenish tones (less mature collagen) and later, reddish tones (more mature collagen). At the interstitium, the fibers become more birefringent as testicular development occurs, showing younger collagen fibers (in green) (Figure 14A–H) which become older in later stages of development (in red) (Figure 14I–L).

### 3.6. Plasma Levels of Sexual Steroids in Synbranchus marmoratus

**Figure 15:**
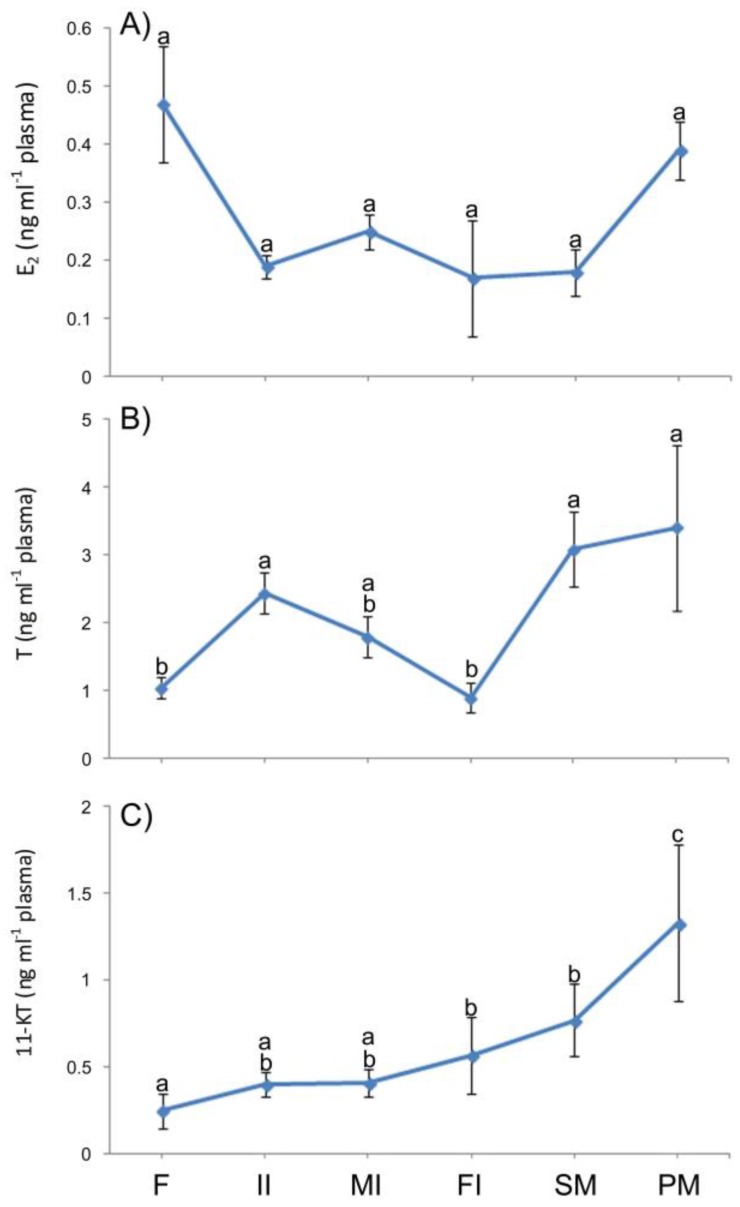
Plasma levels of: (**A**) 17β-Estradiol (E2); (**B**) Testosterone (T); and (**C**) 11-Ketotestosterone (11-KT) (mean ± S.E.M) in the different sex types of *S. marmoratus* individuals. F: females (*n* = 49), II: Initial intersexes (*n* = 67), MI: Mid intersexes (*n* = 35), FI: Final intersexes (*n* = 4), SM: secondary male (*n* = 24), PM: primary male (*n* = 7). Different letters denote significant differences (*p* < 0.05).

Plasma levels of 17β-estradiol (E2) showed no significant difference between classes of individuals (females, initial intersexes, mid intersexes, final intersexes, secondary male, and primary male) (Figure 15A). However, there was a clear decline from 0.47 ± 0.10 ng mL^−1^ (in females) to 0.19 ± 0.02 ng mL^−1^ (initial intersexes), remaining low until the end of sex reversal (secondary males). Levels of 17β-estradiol were very high in primary males (039 ± 0.05 ng mL^−1^) compared to secondary males (0.18 ± 0.04 ng mL^−1^).

Plasma levels of testosterone (T) increased significantly from 1.04 ± 0.16 ng mL^−1^ (females) to 2.44 ± 0.30 ng mL^−1^ (initial intersexes), decreasing significantly and progressively until the end of the transitional process (final intersexes) (Figure 15B). The maximum plasma levels of testosterone were detected in primary and secondary males (3.41 ± 1.22 and 3.09 ± 0.56 ng mL^−1^, respectively).

Regarding 11-ketotestosterone levels (11-KT), there was an increase from 0.40 ± 0.10 ng mL^−1^ (females) to 0.57 ± 0.07 ng mL^−1^ (final intersexes). The maximum plasma levels of 11-ketotestosterone were also detected in primary and secondary males (1.33 ± 0.45 and 0.77 ± 0.21 ng mL^−1^) (Figure 15C).

## 4. Discussion

### 4.1. Gonadal Remodeling

The mechanism of sex reversal in *Synbranchus marmoratus* is characterized by the degeneration of the gonadal tissue corresponding to the first sex, followed by the substitution, development, and maturation of the germinal tissue of the opposite sex, having a period corresponding to an intersex phase, as seen in other species [5,10,11,12,17,40]. Moreover, in the morphological aspects, as herein reported, these events that result in the reversion of the sex in the adult animals are quite similar to those observed in the juvenile hermaphroditism [17].

This process shows the high bipotentiality of germ cells, as stem cells. Morphologically similar in both sexes, the primordial germ cells remain undifferentiated until they are exposed to external factors and hormonal influences that induce their differentiation into oogonia or spermatogonia [5]. In the present study, during the formation of secondary males, primordial germ cells initially differentiate into oogonia for development of an ovary. However, some of them may remain quiescent during gonadal development of the first sex and later, through various stimuli, become active, differentiating into spermatogonia and forming the male gonadal tissue. This same process occurs, for example, in juvenile individuals who have their sexual development altered by social or exogenous factors. In all cases, these animals exhibit enormous plasticity in their sexual patterns [1]. This is the case, for example, of adults of *Oryzias latipes* [41], which present undifferentiated gonia in the adult male gonadal tissue, which can be induced to differentiate into oogonia, giving rise to an ovary through the administration of steroids [42].

Therefore, regardless of the type of stimulus (natural or artificial), the formation of a new gonad during sequential hermaphroditism in adults is nothing more than a gonadal neodifferentiation that follows the same mechanisms found during the period of gonadal morphogenesis of juvenile individuals [17,40]. In addition to these factors, the infiltration of large quantities of granulocytes into the gonad to be remodeled, and the structural disorganization and the proliferation of Leydig cells coinciding with the beginning of the gonadal remodeling process in sequential hermaphroditism reinforces the idea of how similar these mechanisms are in a juvenile [17] and adult animal (present study). In *Sparus aurata*, for example, the infiltration of acidophilic granulocytes is essential for formation and remodelling of the testicular tissue during the gonadal differentiation [43]. In adults of *S. aurata*, an infiltration of these granulocytes is also observed at post-spawning. This event, associated with the role of MMPs, allows the regulation of the testicular physiology and the organization of the cysts during spermatogenesis and post-spawning [44].

### 4.2. The Basement Membrane in the Remodeling of the Germinal Epithelium

In *Synbranchus marmoratus*, the basement membrane was detected by the Reticulin Method at all stages of the sex reversal process, even during the intense gonadal remodeling. Considering *Gymnocorymbus ternetzi* [17], as the only example in which there is a study on the presence/absence of the basement membrane during hermaphroditism, *S. marmoratus* presents a marked difference, as regards the basement membrane around the ovarian follicles, even during the intersex. In the ovarian follicles of transitional gonads of *G. ternetzi*, the basement membrane is completely absent, even in remaining oocytes of completely developed testes [17]. However, these oocytes are stationary in primary growth, so the absence of the basement membrane should be responsible for preventing its development. Contrary data found in *S. marmoratus* may reinforce this idea that the basement membrane is decisive for the development of the ovarian follicle, since the oocytes of *S. marmoratus*, enveloped by a basement membrane always present, continue their development, entering secondary growth, and are capable of reaching final maturation.

The basement membrane is a thin sheet of extracellular matrix that separates the epithelial cells from the subjacent connective tissue. Among its many functions, it participates in the process of cell proliferation, establishes cellular polarity, induces cellular differentiation, and constitutes a structural barrier against the invasion of undesirable cells [45]. Therefore, it is responsible for the maintenance of tissue integrity and preventing inappropriate cell death [46].

When synthesized in the fish ovary, the basement membrane supports the epithelium and surrounds the ovarian follicles, allowing the development of the oocytes [47]. In the fish testis, the basement membrane is synthesized by Sertoli cells, and is thus intimately associated with the establishment of the blood testis barrier. In addition, the integrity of the basement membrane provides conditions for the nutrition, differentiation, and maintenance of the male germ cells, leading to the consequent development of the male germinal epithelium [48,49,50,51].

Despite the basement membrane being present in the transitional gonads of *Synbranchus marmotarus*, it becomes quite sinuous at this stage. Concomitantly, the oocytes enter into atresia and there is an invasion of a large amount of granulocytes in the gonadal tissue. The lack of a physical structure for the basement membrane of the ovary follicles occurs in the first events that signal the end of each reproductive cycle during the fish life [52]. Similarly, cell proliferation, differentiation, and migration, as well as different penetration by stroma, are crucial events for sexual differentiation of the gonads observed in *Xenopus laevis* during its development [53]. So, it is plausible to assume that this folding of the basement membrane results from the punctual lack of components of the extracellular matrix, caused by remodeling of the tissue, when the metalloproteinases become active.

### 4.3. Expression of the Matrix Metalloproteinases Enzymes during the Sex Reversion

Until now, there has been no data on the detailed formation of the basement membrane and of the interstitial compartment remodeling. Similarly, there is no information on the action of the matrix metalloproteinases in the sex reversal process in hermaphroditic species, as in the case of the animal model of this study, *Synbranchus marmoratus*.

The extracellular matrix regulates the basic processes in the tissues and their maintenance through the action of metalloproteinases (MMPs) that contribute to movement, growth, differentiation, and cellular surviving [54]. MMPs have an important role in the maintenance of the tissue of vertebrates, among them, the fish, as verified in this study, in the remodeling of the gonads. In vertebrates in normal conditions, lots of the metalloproteinases are considered a constitutive component of fibroblasts, epithelial, and endothelial cells, being expressed according to the need of the tissue in which they are [55].

In mammals, the MMP-9 and MMP-12 metalloproteinases, with gelatinolitic activity, participate as regulators of events that promote alterations in the male gonads, as well as being expressed in Sertoli and germ cells, participating in events of remodeling of the germinal epithelium [24].

In Teleostei, data about the action of metalloproteinases (MMPs) in the gonads are scarce; however, Chaves-Pozo and collaborators [44] found that those MMPs are expressed both by the germ cells and the somatic cells during the male and female reproductive cycle. Studies on the action of MMPs in males of Teleostei were also described by Santana and Quagio-Grassiotto [16]. These authors suggest that the fibroblasts may be the major cells responsible for the production and secretion of MMP-2 in the interstitial compartment of the catfish *Pimelodus maculatus* (Siluriformes: Pimelodidae), actively participating in the alterations that occur in the gonad during the reproductive cycle.

In this aspect, the changes in the interstitial compartment of the gonads of Teleostei are even more intense in animals that present sex reversal, in which there is an enormous remodeling of the gonadal tissue, especially in the interstitial components, which break a previous structure to reconstruct a new gonadal architecture, formed by the opposite sex gonad. In this study, this gonadal remodeling in adults of *Synbranchus marmoratus*, during the sequential hermaphroditism, was the result, at least partially, of the action of the MMP-2 and MMP-9 metalloproteinases, activated by a third transmembrane metalloproteinase, MMP-14 (also known as MT1-MMP). The three distinct types of MMPs were detected during the gonadal remodeling and produced by different cellular types and not only by fibroblasts.

In the initial stage of the process of gonadal remodeling in *Synbranchus marmoratus*, MMP-9 was detected, especially in the interstitial tissue, in gonads in the early transitional phase. However, during the intermediary transitional phase of the gonadal tissue, the oocytes significantly expressed the enzyme MMP-9. In females that did not undergo the gonadal remodeling process, the expression of MMP-9 was detected sporadically in stromal ovarian cells and never in the oocytes. This shows an active role of the oocytes in the gonad remodeling process. MMP-2 is expressed in male germline cysts, somatic cells, interstitium, and granulocytes. Its action decreases at the end of the sex reversal process, being more active during the intersex stage. After the total gonadal sex reversal, MMP-2 was only detected in granulocytes, similar to MMP-9. Given the above, it seems to be a relation between the activity of the MMPs and the momentary constitution of the gonadal tissue. That is, in the beginning of the remodeling process, when there is a prevalence of female gonadal tissue, MMP-9 is highly expressed, especially in the oocytes. At the end of the process, when the male gonadal tissue is predominant, MMP-2 is more active, especially in germline cysts and granulocytes. Other data obtained in this study, which sustains the hypothesis of the MMP-2 metalloproteinase having a significant participation in the remodeling of the male gonad, shows that this enzyme is intense during the development of the testicular lobules of primary males of *S. marmoratus*.

MMP-9 and MMP-14 metalloproteinases also presented intense expression in this stage, which is characterized by intense proliferation of spermatogonia, thus expanding the germinal epithelium, as well as widening the luminal compartments of the lobules. After the total development of the testis, none of these MMPs were detected in the stages analyzed. However, it is implicit that, if the male reproductive cycle is followed, similar data to those of Santana and Quagio-Grassiotto [16] will be obtained, since the granulocytes of fully developed testes of *Synbranchus marmoratus* showed a positive response to MMP-9. That is, its action and expression should vary according to different phases of the reproductive cycle.

The expression and co-localization of the MMP-14 metalloproteinase frequently coincided with the expression of the other MMPs studied here. Possibly, the correlation of this fact occurs because it is a transmembrane protein that activates the other MMPs, so that they can act on determined tissues [20].

Considering that the metalloproteinases (MMPs) are expressed in different cell types and in different phases of the gonadal remodeling, it is possible to think that, initially, the cells from the germinal epithelium start the production of MMPs, promoting the break-up of the basement membrane, leading to further infiltration of granulocytes, which in turn also participate actively in this process. The absence of the basement membrane can lead to cell death of part of the germinal epithelium, which would lose the support and nutrition by the basement membrane. In this way, several events occur by the action of MMPs: reorganization of the extracellular matrix; reestablishment of the germ and somatic cells, followed by the formation of a new basement membrane; and reconstruction of new gonadal tissue.

### 4.4. The Sexual Steroids and the Expression of the 3β-Hydroxyterid Dehydrognase (3β-HSD) during the Sex Reversal

The detection of the activity of enzyme 3β-hydroxysteroid dehydrogenase (3β-HSD) is used as a universal marker for Leydig cells [56]. The 3β-HSD enzyme is involved in several steps of the biosynthesis of steroid hormones, being responsible for the conversion of pregnenolone to progesterone; 17α-hydroxypregnenolone to 17α-hydroxyprogesterone; dehydroepiandrosterone to androstenedione; and androstenediol to testosterone [26,57]. Thus, it can be assumed that, at the time of its detection, plasma levels of testosterone should be high or at least present.

In this aspect, studies on gonadal differentiation in Teleostei show that the expression of 3β-HSD seems to be related to the onset of spermatogenesis [41]. However, they may be expressed early in male gonads with newly established germinal epithelium [58]. Thus, during gonadal differentiation, in gonads that undergo gonadal remodeling during sex reversal, 3β-HSD expression can be detected in interstitial cells, precursors of Leydig cells [17]. These cells also have a high proliferation rate, detected by immunohistochemistry for PCNA (proliferating cell nuclear antigen). These data are exactly the same as those found in the present study for adults of *Synbranchus marmoratus*, in which this cell type is also found forming cell clusters. Therefore, this data is clear evidence that these cells are involved in gonadal remodeling, helping in the process of construction of the new male gonad.

In relation to plasma levels, the lowest levels of testosterone (T) were found in females (1.04 ng mL^−1^) and in animals in the final transitional phase (0.9 ng mL^−1^) of *Synbranchus marmoratus*. In both primary and secondary males, the levels are quite high (3.41 and 3.09 ng mL^−1^, respectively). An interesting finding is a peak of2.44 ng mL^−1^ in animals at the beginning of transition, which showed a quite high positive response for detection of the 3β-HSD enzyme.

Plasma levels of 11-ketotestosterone (11-KT) in *Synbranchus marmoratus* always increased during sex reversal, from 0.4 ng mL^−1^ in the early transitional gonad, reaching 0.77 ng mL^−1^ in the secondary males. Thus, both testosterone and 11-KT showed increasing levels during sex reversal. In the Teleostei, both hormones are found in high levels in male adults, with 11-KT being responsible for the development of the testis, proliferation of the spermatogonia, final maturation of the gonad, and maintenance of the spermatogenesis [5,59,60]. These statements reinforce the events that occurred during the process of gonadal remodeling, with the female gonad in degeneration and development of new gonadal tissue, now a male gonad.

The variations in the plasma levels observed in the females in an early transitional gonad, with the increase of 17β-estradiol (E2) and decrease of testosterone (T) levels, can be associated with the aromatization of T, which leads to the synthesis of E2 [15,61].

## 5. Conclusions

The extracellular matrix components of the interstitial tissue of the gonads of the *Synbranchus marmoratus* are remodeled through the metalloproteinases (MMPs) according to morphophysiological alterations that occur during sex reversal. This renewal of extracellular matrix components is orchestrated by both cells from germinal and interstitial compartments. However, the breakdown of the basement membrane is the trigger to initiate the remodeling in both compartments and this is due to the action of MMPs, produced by different cell types. These data are confirmed by the constant renewal of the collagen fibers during the sex reversal process in *S. marmoratus*.

Helping in the sex reversal process, sex steroids begin to be synthesized as the proliferation and differentiation of Leydig cells occurs. At the same time, the remodeling of the gonadal tissue begins until it reaches total sex reversion. During the process, the metalloproteinases (MMPs) are expressed, promoting this remodeling, and consequently, the collagen fibers are replaced by new connective tissue elements. Thus, the synthesis of steroids coincides with gonadal remodeling, as the MMPs are synthesized by the cells of the germinal epithelium, as well as the interstitial tissue.

Using an excellent biological model (a hermaphroditic fish, which presents naturally sex reversion), this study will contribute significantly to the development of new techniques based on the inhibition and/or activation of the MMPs, providing new perspectives in experimental studies involving remodeling of the extracellular matrix in many different areas of the biology of the reproduction of fish, such as gonadal differentiation, the reproductive cycle, life history, and sex manipulation.

## Supplementary Materials

The following are available online at: http://www.mdpi.com/2073-4409/7/5/34/s1. Figure S1. Cross section of the gonad of *S. marmoratus* showing melanomacrophage centers and granulocytes. Figure S2. Negative control of the immunohistochemistry for detection of the metalloproteinases in gonads of *S. marmoratus*. Figure S3. Positive control of the immunohistochemistry for detection of the metalloproteinases in ovary of mouse (Swiss). Figure S4. Activity of the gelatinases (MMP-2 and MMP-9) in the gonadal tissue of the *S. marmoratus* (ovary and transitional ovary during intersex). Figure S5. Activity of the gelatinases (MMP-2 and MMP-9) in the gonadal tissue of the *S. marmoratus* (testis of secondary and primary male).
