# Supplementary material for: Action of the Metalloproteinases in Gonadal Remodeling during Sex Reversal in the Sequential Hermaphroditism of the Teleostei Fish Synbranchus marmoratus (Synbranchiformes: Synbranchidae)
Source: Cells. 2018 Apr 24;7(5):34. doi: 10.3390/cells7050034 (PMC5981258; doi:10.3390/cells7050034)
Supplement: Supplementary file 1 [file cells-07-00034-s001.pdf]

SUPPLEMENTARY MATERIAL

Sample preparation for light microscopy

To characterize the granulocytes and melanomacrophage centers, cross sections were prepared for light microscopy, as described in Material and Methods (Figure S1A-E,G-I).

For negative e positive control of the Immunohistochemistry for metalloproteinases, cross sections of the gonads of *Synbranchus marmoratus* (negative control – Figure S2) and ovaries of the mice – swiss (positive control – Figure S3) were prepared for Immunohistochemistry as described in Material and Methods.

Transmission electron microscopy

For electron microscopy, the gonadal tissue of *Synbranchus marmoratus* was post-fixed for 2 h in the dark in 1% osmium tetroxide (in the same buffer). To highlight the cellular structures, block-staining was carried out using an aqueous solution of 5% uranyl acetate for 2 h. Subsequently, the specimens were dehydrated and embedded in Araldite, sectioned, and post-stained with a saturated solution of uranyl acetate in 50% ethanol and 0.2% lead citrate in NaOH (1 N). Electron micrographs were obtained using a Tecnai Spirit Fei Company Transmission Electron Microscope. Transmission electron microscopy was used to characterize granulocytes and melanomacrophage centers (Figure S1F,J).

In situ zymography

For localization of gelatinolytic activity, in situ zymography was performed according technique proposed by Hadler-Olsen and collaborators with minor modifications. The gonads were fixed in 36.7 mM $ZnCl_2$, in 0.1 M Tris, pH 7.4 for 12h before dehydration and Paraplast® (Sigma-Aldrich) embedding. The sections were heated at 59ºC overnight, deparaffinized in xylene, and rehydrated in graded alcohol baths. Substrate was prepared by dissolving 1 mg DQ gelatin in 1.0 ml Milli-Q water, and this was further diluted 1:50 in a reaction buffer containing 50 mM Tris-HCl, 150 mM NaCl, 5 mM $CaCl_2$, and 0.2 mM sodium azid (pH 7.6). Of this mixture, 250 ml was put on top of tissue sections, covered with Parafilm, and incubated in a dark humidity chamber at 37ºC. After 2 h, the Parafilm was removed, and the sections were rinsed with Milli-Q water and fixed in 4% neutral-buffered formalin for 10 min in the dark. Sections were then rinsed in PBS baths (5 min) and mounted with Fluoroshield Mounting Medium® with DAPI (Sigma-Aldrich). To verify the contribution of metalloproteinases, control slides were pre-incubated with 20 mM EDTA for 1 h. 20 mM EDTA was also added to the substrate. Sections were analyzed using fluorescent microscope (BX61, Olympus). Equivalent sections were stained with Toluidine Blue (TB) to observe the gonadal morphology (Figure S4, S5).

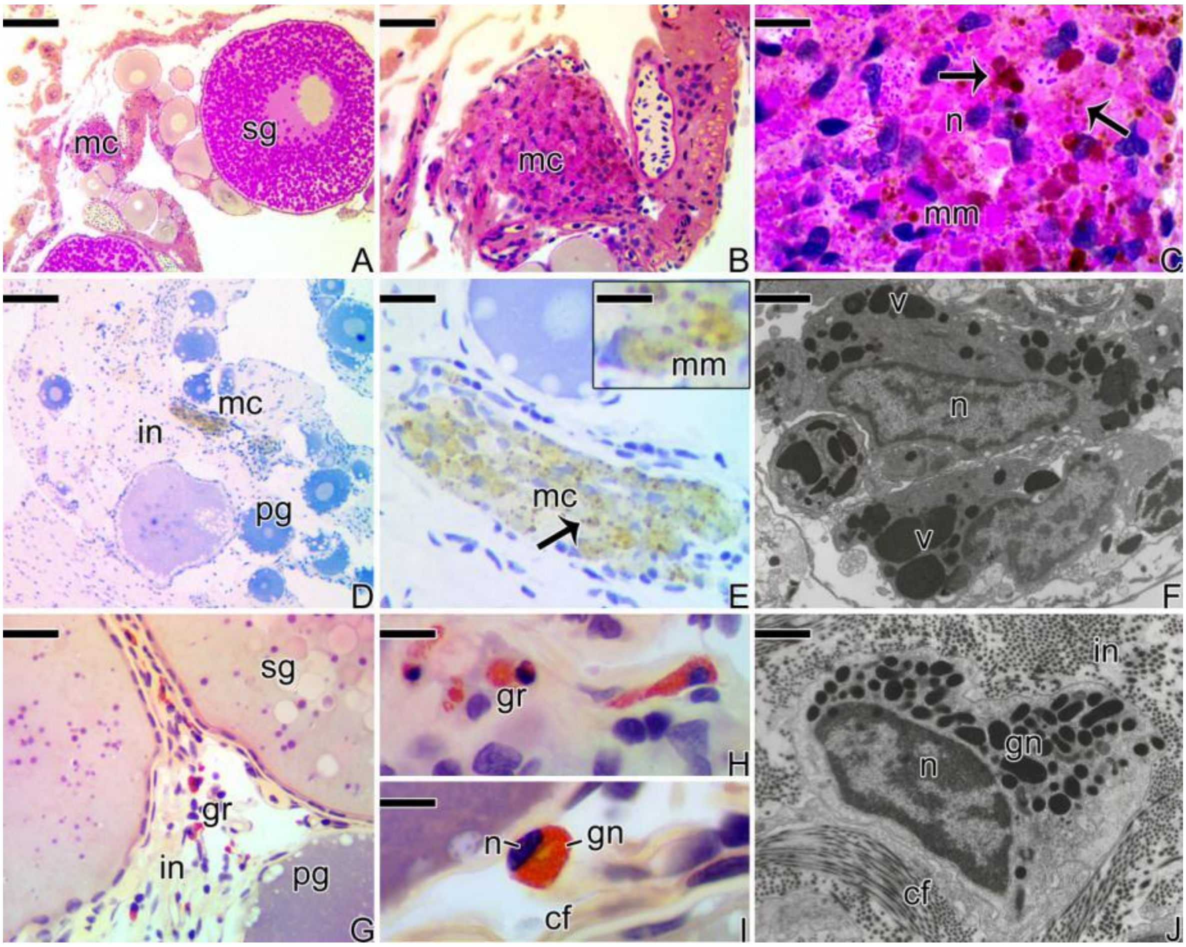

Figure S1. Cross section of the gonad of *S. marmoratus* showing melanomacrophage centers and granulocytes. A-E) Transitional ovary during intersex showing the presence of melanomacrophage centers. B-C) Detail of the melanomacrophage center of A. Note the melanin granules in the cytoplasm of the melanomacrophage cell. D-E) Melanomacrophage center. E-inset) Note the natural pigmentation of the melanomacrophage cell due to presence of the melanin granules. F) Transmission electron microscopy of the melanomacrophage center. Note the eletron-dense vesicles. G-I) Transitional ovary during intersex showing the presence of granulocytes in the interstitial compartment. H-I) Details of G showing granules of the cell. J) Transmission electron microscopy of a granulocyte in the interstitial compartment. Note the eletron-dense granules. Melanomacrophage center (mc), secondary growth oocyte (sg), nuclei (n), melanin granules (arrow), interstitium (in), melanomacrophage cell (mm), eletron-dense vesicle (v), primary growth oocyte (pg), granulocyte (gr), granules (gn), collagen fibers (cf). Counterstaining: MY (A-C,G-I), TB (D-E-inset), Transmission electron microscopy (F,I). Bar: 100μm (A,D), 10μm (B,E), 5μm (C,E-inset), 0.7μm (F,J).

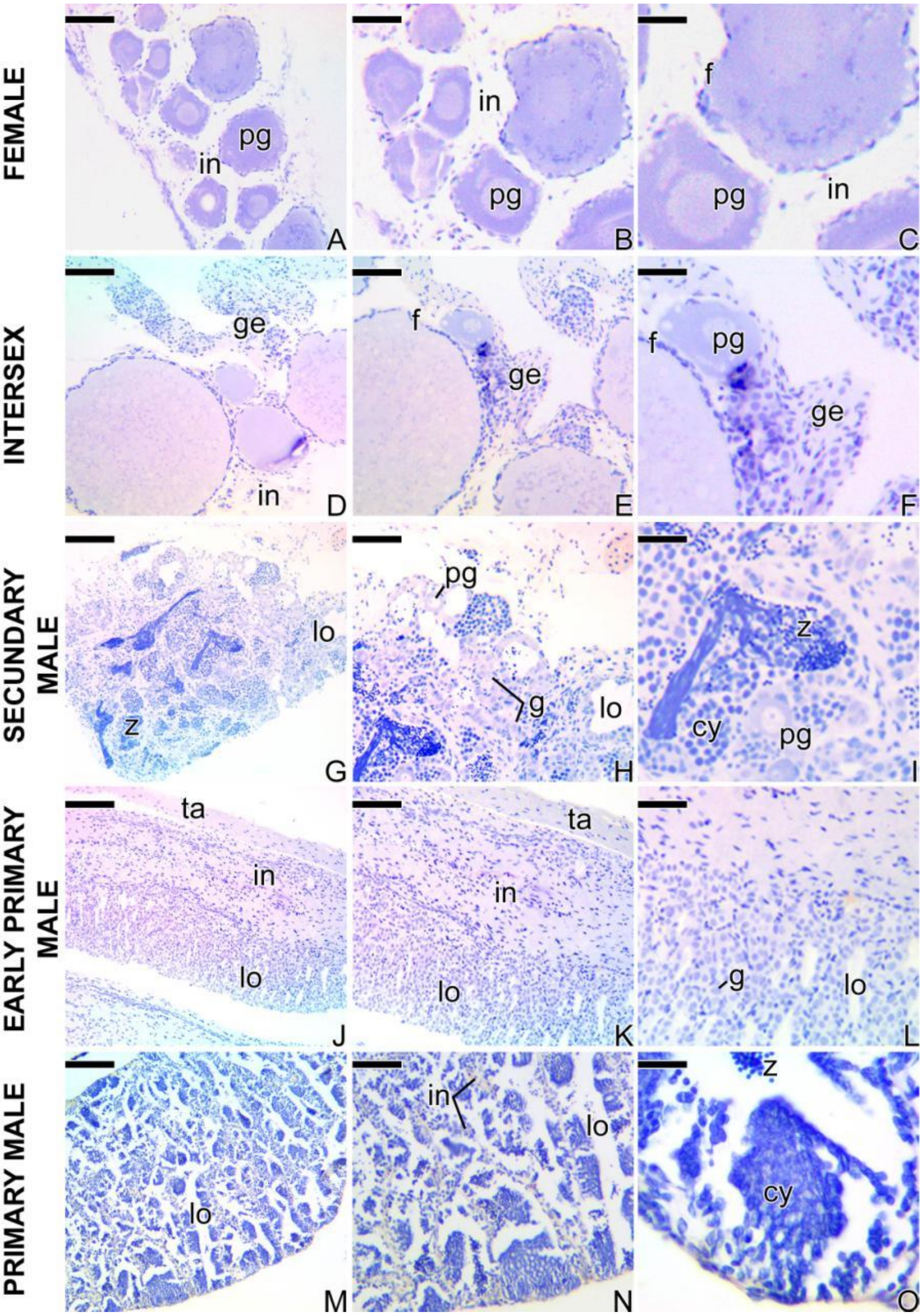

Figure S2. Negative control of the immunohistochemistry for detection of the metalloproteinases in gonads of *S. marmoratus*: A-C) Ovary. D-F) Early transitional ovary during intersex. G-I) Testis of the secondary male. J-L) Testis of the early primary male. M-O) Testis of primary male. Primary growth oocyte (pg), interstitium (in), follicle cells (f), germinal epithelium (ge), lobule (lo), spermatozoa (z), spermatogonia (g), cyst (cy), tunica albuginea (ta). Counterstaining: Harrys Hematoxylin. Bar: 100µm (A,D,G,M), 50µm (B,E,H,J,N), 20µm (C,F,I,K,O), 10 µm (L).

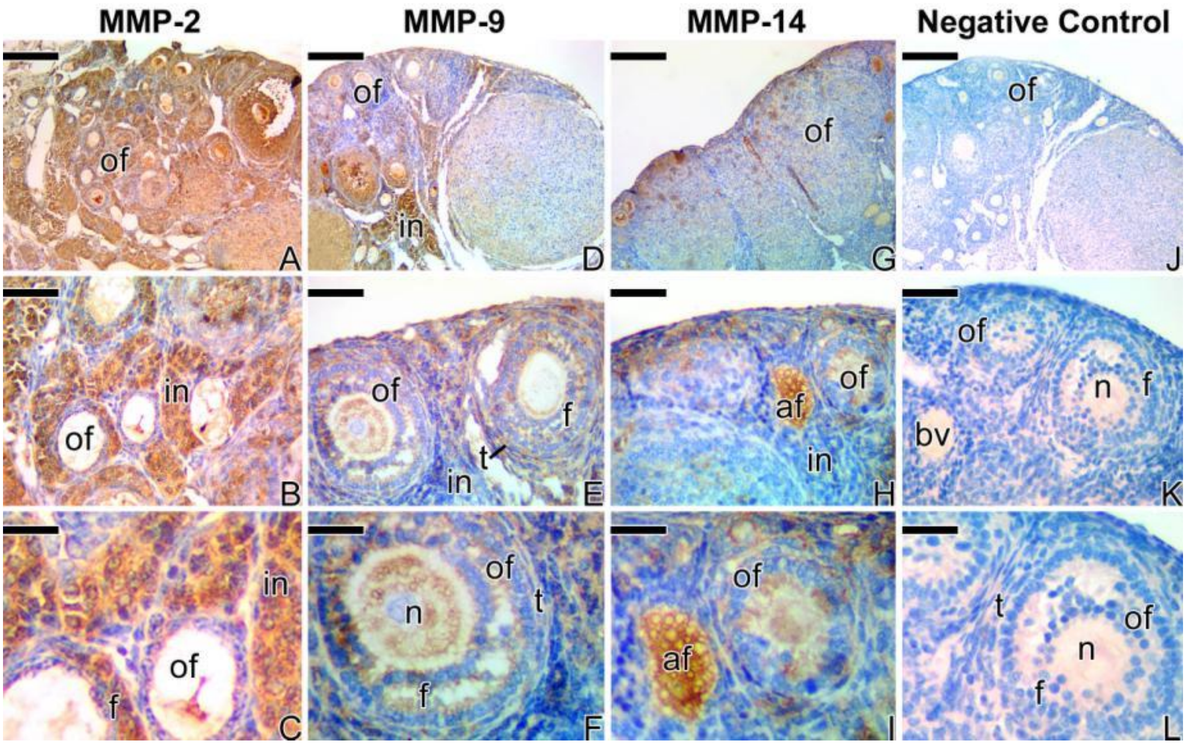

Figure S3. Positive control of the immunohistochemistry for detection of the metalloproteinases in ovary of mouse (Swiss). A-C) Immunolocalization of the MMP-2. D-F) Immunolocalization of the MMP-9. G-I) Immunolocalization of the MMP-14. J-L) Negative control. Ovarian follicle (of), interstitium (in), follicle cells (f), theca (t), atretic follicle (af), blood vessel (bv), nuclei (n) Counterstaining: Harrys Hematoxylin. Bar: 100µm (A,D,G,L), 50µm (B,E,H,K), 20µm (C,F,I,L).

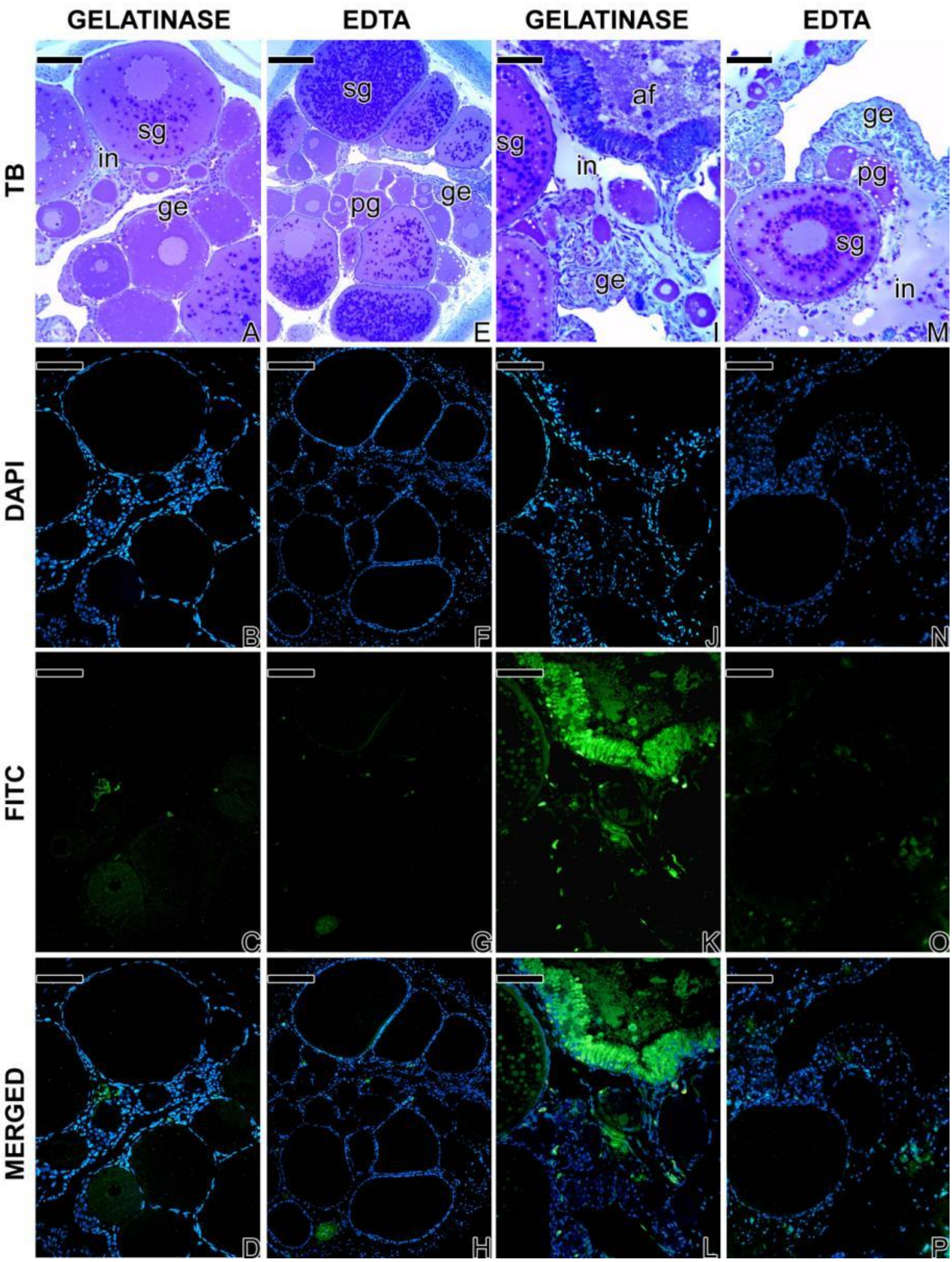

Figure S4. Activity of the gelatinases (MMP-2 and MMP-9) in the gonadal tissue of the *S. marmoratus* (ovary and transitional ovary during intersex). A,E,I,M) Cross section of the gonadal tissue stained with Toluidine Blue (TB). B-D, F-H, J-L, N-P) In situ zymography of gonadal tissue of the *S. marmoratus* using DQ-gelatin substrate. Green fluorescence (FITC) shows gelatinolytic activity, whereas nuclei are shown in blue (DAPI). A-H) ovary. I-P) Transitional ovary during intersex. E-H, M-P) To demonstrate the contribution from MMPs, 20 mM EDTA was added to the substrate (negative control). Secondary growth oocyte (sg), germinal epithelium (ge), interstitium (in), primary growth oocyte (pg), atretic follicle (af). Bar: 100μm (A-D), 130μm (E-H), 70μm (I-P).

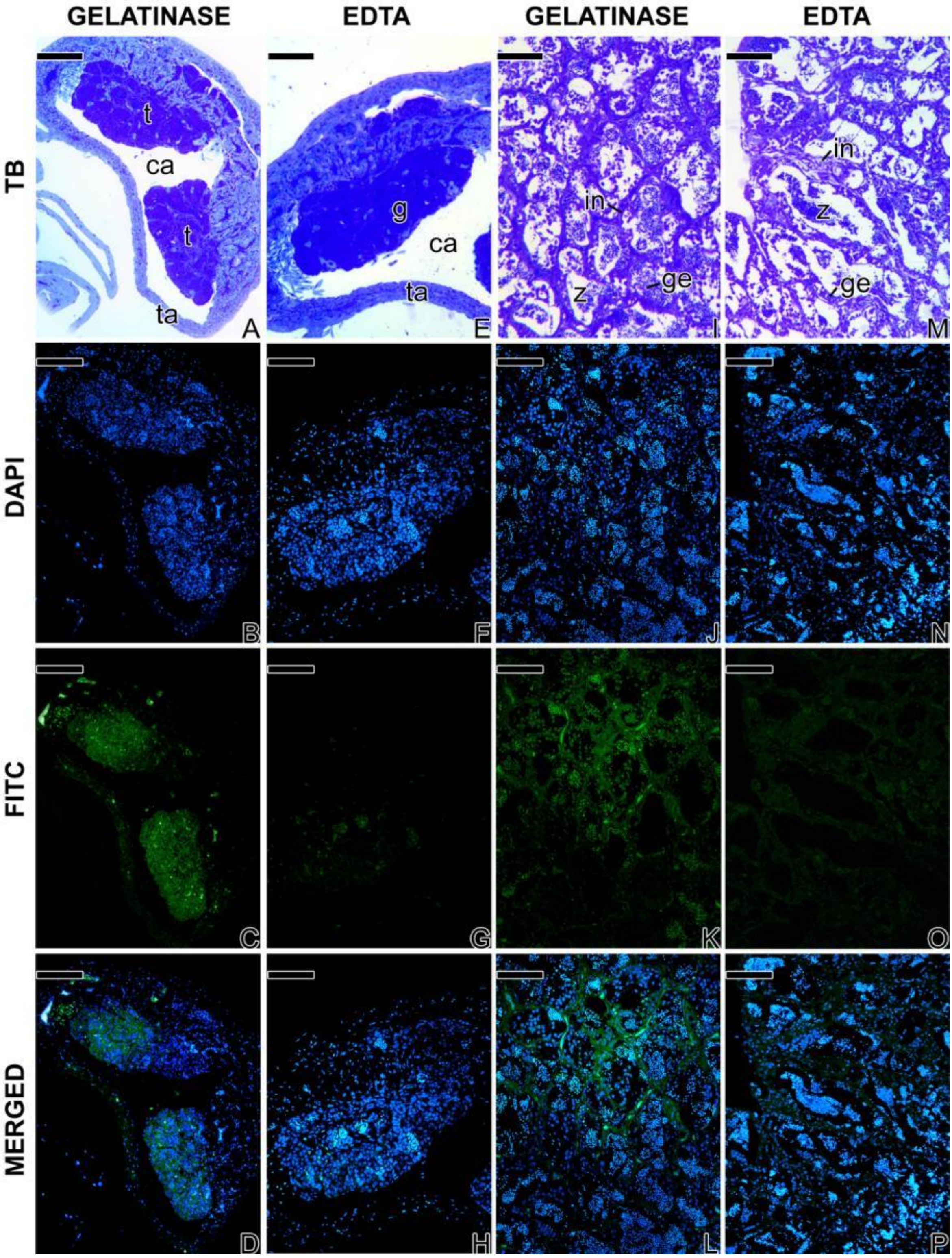

Figure S5. Activity of the gelatinases (MMP-2 and MMP-9) in the gonadal tissue of the *S. marmoratus* (testis of secondary and primary male). A,E,I,M) Cross section of the gonadal tissue stained with Toluidine Blue (TB). B-D, F-H, J-L, N-P) In situ zymography of gonadal tissue of the *S. marmoratus* using DQ-gelatin substrate. Green fluorescence (FITC) shows gelatinolytic activity, whereas nuclei are shown in blue (DAPI). A-H) testis of secondary male. I-P) testis of primary male. E-H, M-P) To demonstrate the contribution from MMPs, 20 mM EDTA was added to the substrate (negative control). Testis (t), ovarian cavity (ca), spermatogonia (g), tunica albuginea

(ta), interstitium (in), germinal epithelium (ge), spermatozoa (z). Bar: 100μm (A-D), 150μm (E-H), 50μm (I-P).
